# Anti-SIA-cIgG enhances chemotherapy effectiveness through PTPN13-regulated tumor stemness in head and neck squamous cell carcinoma

**DOI:** 10.1515/jtim-2026-0040

**Published:** 2026-03-26

**Authors:** Luming Wang, Yangyang Xia, Chenshu Liu, Xiaofeng Shan, Yi Sui, Shang Xie, Zhigang Cai

**Affiliations:** Department of Oral and Maxillofacial Surgery, Peking University School and Hospital of Stomatology; National Center for Stomatology; National Clinical Research Center for Oral Diseases; National Engineering Research Center of Oral Biomaterials and Digital Medical Devices; Beijing Key Laboratory of Digital Stomatology; NHC Key Laboratory of Digital Stomatology; NMPA Key Laboratory for Dental Materials, Beijing, China

**Keywords:** SIA-cIgG, head and neck squamous cell carcinoma, chemoresistance, patient-derived organoids, PTPN13, tumor stemness

## Abstract

**Background and Objectives:**

The chemotherapy response rate in head and neck squamous cell carcinoma (HNSCC) remains low due to a lack of effective therapeutic targets, and treatment efficacy is further limited by chemoresistance and heterogeneity in drug response. Sialylated cancer IgG (SIA-cIgG) is a tumor-derived immunoglobulin implicated in tumor stemness. However, the relationship between SIA-cIgG and chemoresistance, and its potential as a therapeutic target, remain to be determined.

**Methods:**

We evaluated the antitumor eficacy of SIA-cIgG inhibition combined with four chemotherapeutic agents using two HNSCC cell lines with high or low SIA-cIgG expression, along with in vivo xenograft models. Furthermore, we investigated the functional roles of SIA-cIgG and its downstream effector PTPN13 in regulating HNSCC stemness. Patient-derived organoids (PDOs) from 25 HNSCC patients were used to compare the antitumor eficacy of anti-SIA-cIgG-based combinations against conventional clinical chemotherapy regimens.

**Results:**

Elevated SIA-cIgG protein levels correlated positively with an increased IC50 for cisplatin and poorer chemotherapy response. SIA-cIgG/PTPN13 axis was critical for tumor stemness and chemoresistance. Anti-SIA-cIgG treatment enhanced PTPN13 protein stability and upregulated PTPN13 mRNA expression via SP1. Anti-SIA-cIgG-based drug combinations demonstrated significantly higher anticancer efficacy than conventional clinical chemotherapy regimens and overcame tumor heterogeneity in drug response.

**Conclusions:**

SIA-cIgG/PTPN13 axis regulates tumor stemness and contributes to chemoresistance, anti-SIA-cIgG-based drug combinations exhibit significant potential for clinical application.

## Introduction

Head and neck squamous cell carcinoma (HNSCC) is the most common pathological type of head and neck malignant tumor, often causing severe functional and aesthetic impairment in the oral and maxillofacial regions.^[1]^ Typically, most patients present with locally advanced or metastatic disease at diagnosis. When the disease progresses to advanced stages with excessive tumor burden or develops recurrent/metastatic lesions unsuitable for surgery, chemotherapy becomes one of the main treatments.^[2,3]^ Conventional chemotherapeutic agents include cisplatin (CDDP), 5-fluorouracil (5-FU), docetaxel (DTX), and paclitaxel (PTX). Advances in molecular targeted therapy and immunotherapy have led to the incorporation of EGFR-targeting agents (*e.g*., cetuximab) and PD-1/PD-L1 inhibitors, as monotherapy or combined with chemotherapy, into clinical guidelines for HNSCC.^[4,5]^ Nevertheless, drug response in HNSCC remains highly heterogeneous. Despite combination chemotherapy and immunotherapy, clinical response rates rarely exceed 40%.^[6, 7, 8, 9, 10]^ Moreover, many patients rapidly develop chemoresistance during treatment, which substantially limits therapeutic efficacy.^[11,12]^ Therefore, there remains an urgent need for highly specific therapeutic targets to overcome current treatment limitations in HNSCC.

Sialylated cancer IgG (SIA-cIgG) is a special type of IgG produced by cancer cells.^[13,14]^ Distinct from B cell-derived IgG, SIA-cIgG is characterized by aberrant N-glycosylation and hypersialylation at the Asn162 residue in the C_H_1 domain, modifications that confer various pro-tumorigenic functions.^[15,16]^ The monoclonal antibody RP215 specifically recognizes and blocks this sialylation site to inhibit SIA-cIgG activity.^[15]^ SIA-cIgG is highly expressed in epithelial squamous cell carcinomas, including lung cancer,^[17]^ breast cancer,^[18,19]^ colon cancer,^[20]^ pancreatic ductal adenocarcinoma,^[21]^ prostate cancer,^[22]^ and head and neck squamous cell carcinoma, *etc*.^[23]^ Elevated expression of SIA-cIgG is positively correlated with poor prognosis and aggressive pathological features. Studies have shown that SIA-cIgG knockdown enhances radiosensitivity in lung adenocarcinoma by suppressing PI3K/AKT/DNA-PKcs pathway activation.^[24]^ Furthermore, SIA-cIgG overexpression is associated with chemoresistance, and its expression levels in fine-needle aspiration biopsy samples can predict pathological response to neoadjuvant chemotherapy in pancreatic ductal adenocarcinoma.^[25]^ Notably, SIA-cIgG plays a critical role in maintaining tumor stemness. Cells with high SIA-cIgG expressions exhibit significantly enhanced sphere-forming ability and patient-derived xenograft (PDX) tumorigenicity than those with low SIA-cIgG expression. SIA-cIgG also activates a self-propagating loop by inducing c-Met-mediated SOX2 expression, which in turn upregulates SIA-cIgG expression.^[26]^ These findings highlight the potential of SIA-cIgG as a therapeutic target. However, its specific role in chemoresistance remains poorly understood, warranting comprehensive exploration and validation.

Therapeutic failure in cancer patients is closely associated with tumor stemness and cancer stem cells (CSCs). Tumor stemness represents a major contributor to intertumoral and intratumoral heterogeneity,^[27]^ with stemness-enriched cell populations demonstrating heightened propensity for multidrug resistance, metastasis, recurrence, eventually leading to treatment failure.^[28]^ Our previous work revealed that knockdown of SIA-cIgG enhanced CDDP-induced tumor cytotoxicity by inhibiting activation of the SRC/ AKT signaling pathway, and identified protein tyrosine phosphatase non-receptor type 13 (PTPN13) as a key mediator of this SIA-cIgG-regulated signaling.^[29]^ PTPN13 is a large phosphatase that acts as a tumor suppressor in several cancers. In hepatocellular carcinoma, PTPN13 is frequently inactivated or lost, and the hepatitis B virus X protein (HBx) suppresses its expression by inducing DNA methylation to promote tumorigenesis.^[30]^ In breast cancer cells, PTPN13 inhibits proliferation and invasion by directly inactivating SRC.^[31]^ In high-grade serous ovarian carcinoma (HGSOC), PTPN13 regulates epithelial-mesenchymal transition and platinum sensitivity.^[32]^ However, whether PTPN13 is involved in regulating tumor stemness, and if the SIA-cIgG/PTPN13 axis contributes to chemoresistance by modulating stemness, remain unknown.

In this study, we demonstrate that SIA-cIgG and its key downstream effector PTPN13 contribute to chemoresistance and stemness maintenance. We further explored the regulatory mechanism by which SIA-cIgG controls PTPN13 expression. Using patient-derived HNSCC organoids (PDOs), we show that anti-SIA-cIgG-based drug combinations are significantly more effective than current clinical regimens, overcoming therapy variability caused by tumor heterogeneity. Collectively, our findings highlight the clinical potential of anti-SIA-cIgG-based drug combinations and elucidate the mechanism of the SIA-cIgG/PTPN13 axis in chemoresistance and stemness.

## Material and methods

### Clinical samples

Clinical samples were obtained from patients who were pathologically diagnosed as HNSCC and underwent surgical treatment or biopsy at the Department of Oral and Maxillofacial Surgery, Peking University School and Hospital of Stomatology, between September 2021 and October 2024. A total of 50 paired samples, including tumor tissues and adjacent normal tissues, were used for immunohistochemistry (IHC) and Western blot analysis. “Adjacent normal tissues” were defined as histologically normal epithelial tissues located approximately 1 cm from the visible tumor margin. Additionally, six sets of samples, including tumor tissues, adjacent normal tissues, and normal tissues, were used for IHC. “Normal epithelial tissues” were defined as histologically normal epithelial tissues located more than 5 cm from the visible tumor margin, obtained during wound trimming and suturing. Tumor tissues from 25 HNSCC patients were used to establish patient-derived organoids (PDOs). Detailed clinical information of the patients is summarized in Supplementary Table S1 and S2. The acquisition and use of all specimens were conducted with informed consent from the patients. Experiments were conducted in accordance with the Declaration of Helsinki and were pre-approved by the Biomedical Ethics Committee of Peking University School of Stomatology (Approval No. PKUSSIRB-202283176).

### Establishment and culture of patient-derived organoids of HNSCC

The establishment and culture of HNSCC PDOs are as previously described,^[33]^ with slight modifications. Briefly, tumor samples obtained during surgery or biopsy are transported on ice to the laboratory and PDO establishment is performed within 24 h. The specimens were washed, minced, and digested with tissue digestion solution (#K601003, BioGenous) at 37 °C and on orbital shaker 100 rpm for 30 min until the supernatant became turbid. Digestion was terminated by adding 2% fetal bovine serum (FBS), and the suspension was filtered through a 70 μm strainer. After two rounds of centrifugation to remove digestive enzymes, cells were mixed with extracellular matrix (#M315066, BioGenous) and seeded in culture dishes. PDOs were cultured in HNSCC organoid medium (#K2152-OSC, BioGenous), which was replaced every three days.

### Medication and organoid cell viability assay

Combination therapies selected for this study were based on National Comprehensive Cancer Network Clinical Practice Guidelines in Oncology—Head and Neck Cancers (Version 5, 2024) and Guidelines of Chinese Society of Clinical Oncology—Head and Neck Cancer (2024).

First-generation (P1) PDOs were used for drug treatment and viability assays. P1 PDOs were seeded at a density of 7500 per well in 96-well plates, with three replicates per condition, and treated with agents for 6 d (the drug-containing medium was replaced on day 3). Drugs used for treatment are as followed: CDDP (#A8321, APExBIO) dissolved in DMF, 5-fluorouracil (#HY-90006, MedChemExpress) dissolved in DMSO, docetaxel (#HY-B0011, MedChemExpress) dissolved in DMSO, paclitaxel (#HY-B0015, MedChemExpress) dissolved in DMSO, cetuximab (#HY-P9905, MedChemExpress), and RP215 (#sc-69849, Santa Cruz). The concentrations of CDDP, 5-FU, DTX, and PTX used in our *in vitro* experiments were selected based on cell IC_50_ values (Fig. S2D-S2K), *In vivo* concentrations were converted from human equivalent doses based on body surface area and were designed to approximate the intensity of clinical regimens (*e.g*., TPF induction) while considering mouse tolerance. The concentration of RP215 was selected with reference to previously published studies.^[26]^ All concentrations were determined through pre-experimental optimization. The duration of chemotherapy were determined based on cell condition and downstream pathway activation observed in preliminary experiments. The time points for the CHX chase assay were selected according to the degradation kinetics of PTPN13 and overall cell condition assessed during pre-experimental optimization. The IC_50_ values of the agents were calculated using GraphPad Prism 9 for Windows (GraphPad Software, Inc.) based on PDOs viability after treatment with serially diluted drug concentrations.

Organoid viability was assessed using the CellTiter-Glo® 3D Cell Viability Assay (#G9683, Promega) according to the manufacturer’s instructions. Briefly, the culture medium was removed, and a 1:1 mixture of the reagent and fresh medium was added to each well. The plates were shaken for 5 min and incubated at room temperature for 25 min, followed by fluorescence measurement using a multimode microplate reader.

For evaluation of PDOs viability and cytotoxicity, Calcein-AM/PI staining (C2015S, Beyotime) was performed. The culture medium was removed, and PDOs were washed with PBS. Each well of a 24-well plate was filled with 500 μL of Calcein-AM/PI working solution and incubated at 37 °C in the dark for 30 min. PDOs were then observed under a fluorescence microscope.

### Cell lines and culture

HNSCC cell line WSU-HN6 (RRID: CVCL_5516) was obtained from the Chinese Academy of Sciences and has been maintained in the Central Laboratory of Peking University School and Hospital of Stomatology. WSU-HN6 is authenticated every six months (latest performed on August 6, 2024). HNSCC cell line SCC-15 (RRID: CVCL_1681) was purchased from the American Type Culture Collection (ATCC) in July 2024. WSU-HN6 was cultured in DMEM (#C11995500BT, Gibco) supplemented with 10% fetal bovine serum (FBS) (#ST30-3302, PAN Biotech), while SCC-15 was cultured in DMEM/F-12 (#C11330500BT, Gibco) medium containing 10% FBS. Both cell lines were maintained at 37 °C in a humidified incubator with 5% CO_2_.

### Immunohistochemistry and immunofluorescence

For IHC, 6 μm-thick frozen sections were incubated with primary antibodies at 4 °C overnight, followed by secondary antibody incubation and DAB staining according to the manufacturer’s protocol (PV6000, ZSGB-BIO). Two trained pathologists independently evaluated the positive staining. The percentage of positive cells was scored as follows: 0 (negative), 1 (< 25% positive cells), 2 (26%–50% positive cells), 3 (51%–75% positive cells), and 4 (> 75% positive cells). The staining intensity was scored as follows: 0 (no staining), 1 (light staining), 2 (medium staining), and 3 (strong staining). The final pathological score was calculated by multiplying the percentage score by the intensity score.

For IF of tumor tissues, samples were incubated with primary antibodies at 4 °C overnight, followed by incubation with FITC- or TRITC-conjugated secondary antibodies at room temperature for 1 h. Nuclei were stained with DAPI, and images were captured using a fluorescence microscope.

For IF of PDOs, PDOs were fixed in 4% paraformaldehyde (PFA) for 1 h, permeabilized with 0.5% Triton X-100 for 20 min, and blocked with 2% bovine serum albumin (BSA) for 30 min. The PDOs were then incubated with primary antibodies at 4 °C overnight, followed by incubation with FITC-conjugated secondary antibodies at room temperature for 1 h. Images were captured using a fluorescence microscope.

All antibodies used in this study are summarized in Supplementary Table S3.

### Cell proliferation, migration, and invasion assays

For cell proliferation, cells were seeded at a density of 6000 cells per well in 96-well plates. When siRNA transfection was required, the transfection system was added simultaneously with cell seeding. After 12 h, drugs were added, and cell viability was assessed 48 h posttreatment using the CCK-8 assay (#CK04, DOJINDO) according to the manufacturer’s instructions. For growth curve analysis, cell viability was measured at 0, 24, 48, and 72 h after seeding, with the 12-h time point set as 0 h.

For cell migration and invasion assays, cells were starved for 12 h, and 1 × 10^5^ cells were resuspended in Opti-MEM (#31985062, Gibco) and seeded into the upper chamber of an 8 μm pore size Transwell insert (#3422, Corning). For the invasion assay, the upper chamber was pre-coated with 10% Matrigel Matrix (#354234, Corning) for 1 h. The lower chamber was filled with DMEM containing 10% FBS. After 24 h, non-migrated or non-invaded cells in the upper chamber were removed, and the migrated or invaded cells were fixed with 4% paraformaldehyde (PFA) and stained with 0.1% crystal violet. Images of six randomly selected fields were captured, and the results were presented as ratio or means ± standard deviation (SD).

### Tumorsphere formation and colony formation assays

For tumorsphere formation assay, WSU-HN6 or SCC-15 cells were resuspended in DMEM/ F12 supplemented with 1% B27 (#17504044, Thermo Fisher Scientific), 1% N2 (#17502048, Thermo Fisher Scientific), 20 ng/mL human recombinant epidermal growth factor (EGF) (#236-EG-01M, R&D Systems), and 10 ng/mL human recombinant basic fibroblast growth factor (bFGF) (#233-FB-025/CF, R&D Systems). The cells were then seeded into ultra-low attachment plates and cultured for 10 d. Spheres with a diameter greater than 70 μm were counted.

For colony formation assay, 250 cells were seeded per well in 12-well plates and cultured for 10 d. Then fixed cells with 4% paraformaldehyde (PFA) for 5 min and stained with 0.1% crystal violet for 10 min. Colonies containing more than 50 cells were counted.

### Flow cytometry for apoptosis detection

Cell apoptosis was detected using the APC Annexin V Apoptosis Detection Kit with PI (#640932, BioLegend). According to the manufacturer’s instructions, adherent cells were prepared as single-cell suspensions, incubated with APC-conjugated Annexin V at room temperature in the dark for 10 min, and then stained with propidium iodide (PI) for 1 min. Apoptotic cells were analyzed using a flow cytometer.

### Transient and stable transfection

Transient transfection of siRNA and plasmids was performed using jetPRIME (#101000046, Polyplus) according to the manufacturer’s protocol. All siRNAs used in this study are summarized in Supplementary Table S4.

For stable knockdown of PTPN13, lentivirus was designed based on the siPTPN13-1 sequence. After viral infection, cells were selected with 1 μg/mL puromycin.

### RNA extraction and quantification

Total RNA was extracted from cells using TRIzol (#15596018, Thermo Fisher Scientific) and SteadyPure RNA Extraction Kit (#AG21024, Accurate Biotechnology). Total mRNA (1 mg) was reverse-transcribed into cDNA using a reverse transcription kit (#RK20429, ABclonal). Quantification was performed using SYBR Green (#RK21203, ABclonal). All primers used in this study are summarized in Supplementary Table S4.

### Co-immunoprecipitation

Cells were lysed using Cell Complete Lysis Buffer (#P0037, Beyotime), and protein concentrations were determined using the BCA protein assay (#23227, Thermo Fisher Scientific). Cell extracts were incubated with primary antibodies at 4 °C overnight with rotation, followed by incubation with magnetic beads (#P2108, Beyotime) at room temperature for 2 h. After adding SDS-PAGE loading buffer (#AQ107, Aoqing Biotechnology), samples were heated at 95 °C for 5 min and then analyzed by Western blot.

### Western blot analysis

The Western blot protocol was performed as previously described.^[29]^ Briefly, total protein was extracted from WSU-HN6 and SCC-15 cells using RIPA buffer (#R0010, Solarbio) supplemented with 1% protease and phosphatase inhibitors (#P002, NCM Biotech). Protein concentrations were determined using the BCA protein assay. Proteins were separated by SDS-PAGE and transferred onto polyvinylidene fluoride (PVDF) membranes (#IPVH00010, Millipore), then blocked with 5% non-fat milk at room temperature for 1 h, incubated with primary antibodies at 4 °C overnight, and then incubated with horseradish peroxidase (HRP)-conjugated secondary antibodies at room temperature for 1 h. All antibodies used in this study are summarized in Supplementary Table S3.

### Chromatin immunoprecipitation

Chromatin immunoprecipitation (ChIP) assay was performed using ChIP Kit (#9003, Cell Signaling Technology). According to the manufacturer’s protocol, cells were cross-linked with 1% formaldehyde for 10 min, collected, and lysed using ChIP lysis buffer. Chromatin was sheared by sonication (250 W, 11 cycles of 6 seconds on and 9 seconds off), and the resulting DNA fragments were immunoprecipitated using an SP1 antibody. The precipitated DNA was quantified by qPCR. All ChIP-qPCR primers used in this study are summarized in Supplementary Table S4.

### Dual-luciferase reporter assay

The PTPN13 promoter region –1500 to +100 bp (NC_000004.12:86593414-86594414) and its mutants (Mutant 1, Mutant 2, Mutant 3) were cloned and inserted into pGL3-Enhancer vector (#212938, Addgene) to construct luciferase reporter plasmids. SCC-15 cells were co-transfected with the reporter plasmids and Renilla luciferase control plasmid (pRL-TK, Promega). Luciferase activities were measured using Dual-Glo Luciferase Assay System Kit (#RG027, Beyotime) following the manufacturer’s instructions. Relative luciferase activity was calculated as the ratio of firefly to Renilla luciferase activities.

### Xenograft mouse model

All animal experiments were conducted in accordance with protocols approved by the Ethics Committee of Animal Research, Peking University Health Science Center (DLASBE0044). Balb/c nude mice (age 5 weeks, female) were obtained from Vital River Laboratories Technology Co., Ltd. and maintained under standard pathogen-free conditions.

Mice were randomly allocated into 16 experimental groups (*n* = 6 per group). Following one week of acclimatization, tumor xenografts were established by subcutaneous injection of 5 × 10^6^ cells per mouse of WSU-HN6 shNC or WSU-HN6 shPTPN13 cells. Treatment initiation began when tumor volumes reached approximately 100 mm^3^. Therapeutic agents were administered via tail vein injection at 4-d intervals for a total of 4 cycles. Tumor volumes and body weights were recorded.

### Data analysis

In this study, HNSCC database from The Cancer Genome Atlas (TCGA) was used for Kaplan-Meier survival analysis and pathway enrichment analysis. Figures were generated using Sangerbox. The correlation between PTPN13 and SP1 mRNA expression in HNSCC was analyzed using GEPIA.

All data were analyzed using GraphPad Prism 9 and SPSS 23.0 (IBM). Each *in vitro* experiment was repeated at least three times, and results are presented as mean ± SD. Comparisons between two independent groups were performed using two-tailed *t*-tests, while comparisons among three or more groups were analyzed using one-way ANOVA followed by Dunnett’s test. *P* < 0.05 was considered statistically significant.

## Results

### High expression of SIA-cIgG is associated with primary CDDP resistance

**Figure 1:**
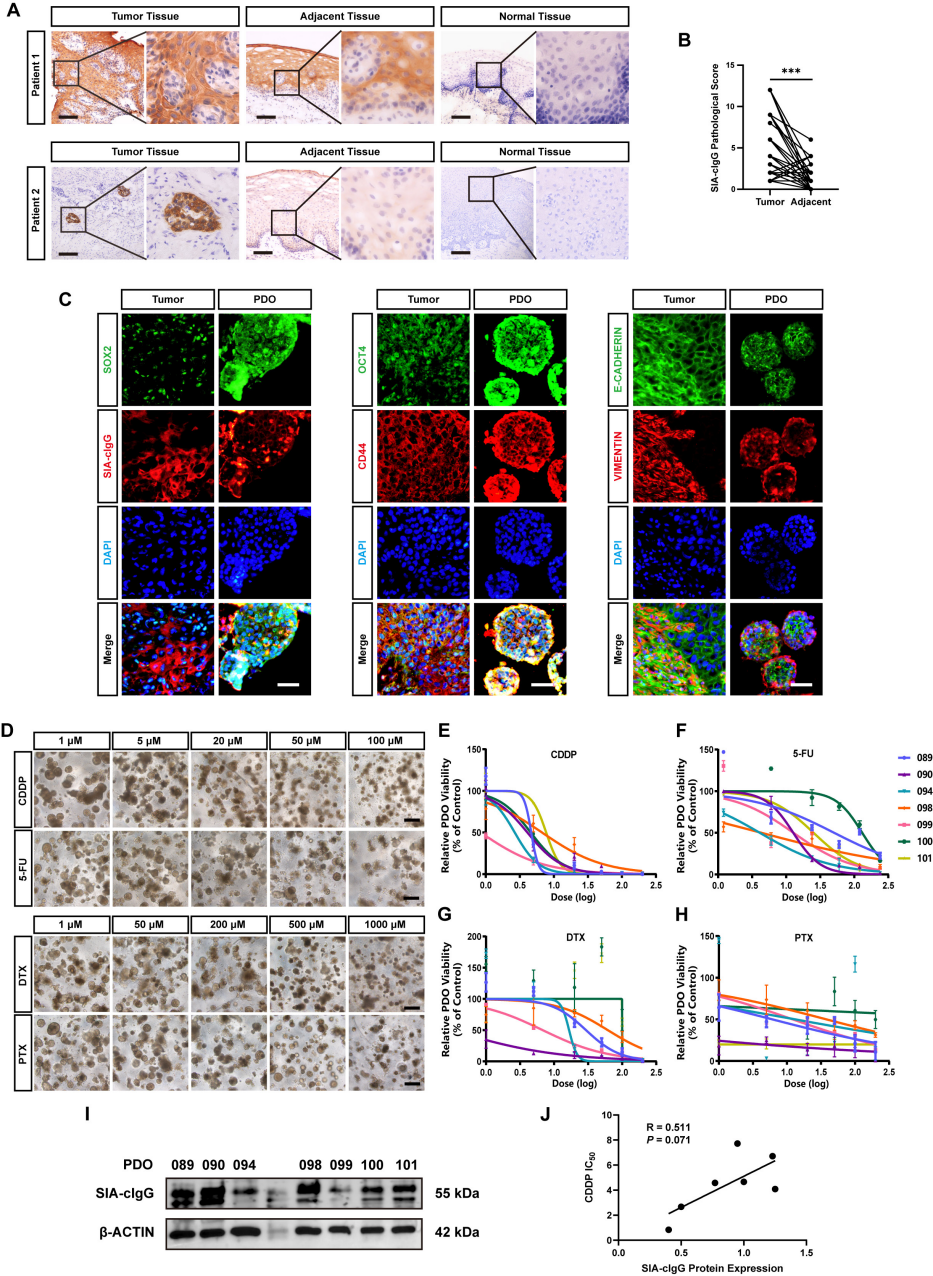
High expression of SIA-cIgG is associated with primary CDDP resistance. (A) Representative microphotographs of SIA-cIgG IHC staining in paired HNSCC tumor tissue, adjacent normal tissue and normal epithelial tissue. Scale bars, 100 μm. (B) Quantification analysis of SIA-cIgG IHC staining in tumor tissue and adjacent mucosal tissue, *n* = 50, ^***^P < 0.001. (C) Representative fluorescence microphotographs of biomarker staining in HNSCC tumor tissue. Scale bar, 100 μm. (D) Representative microphotographs of HNSCC PDOs after gradient concentration treatments of CDDP, 5-FU, DTX and PTX. Scale bars, 250 μm. (E-H) Drug response curves of CDDP (E), 5-FU (F), DTX (G) and PTX (H) in 7 PDOs. (I) SIA-cIgG protein expression of 7 PDOs by Western blot assay. (J) Simple linear regression of SIA-cIgG protein expression and CDDP IC50, *n* = 7.

SIA-cIgG is widely expressed in epithelial cancer cells, with subcellular localization observed in both cytoplasm and cell membrane,^[16,26]^ supporting its potential as a therapeutic target. In this study, we evaluated SIA-cIgG expression by immunohistochemistry (IHC) in 50 pairs of HNSCC tumor tissues and matched adjacent normal tissues, as well as in normal epithelial tissues from 6 HNSCC patients. Representative IHC staining of SIA-cIgG in paired tumor, adjacent, and normal samples are presented in Figure 1A. IHC analysis confirmed that SIA-cIgG expression was observed in all tumor tissues. Among the 50 matched adjacent non-tumor tissues, 29 exhibited positive staining, while none of the normal tissue samples were positive. Overall, SIA-cIgG expression levels were significantly higher in tumor tissues compared to adjacent tissues (Figure 1B).

Patient-derived organoids (PDOs) are preclinical models that faithfully recapitulate the drug response profiles of the original tumors.^[34,35]^ We established HNSCC PDOs, which retained the expression patterns of SIA-cIgG, stemness markers (CD44,OCT4 and SOX2), and EMT markers (E-CADHERIN and VIMENTIN) found in the parental tumor tissues (Figure 1C). To investigate the relationship between SIA-cIgG expression and chemosensitivity, and to assess intertumoral variability in drug response, we treated 7 PDOs with concentration gradients of four commonly used chemotherapeutics: CDDP, 5-FU, DTX, and PTX (Figure 1D). Dose-response curves were plotted based on viability to determine IC_50_ values (Figure 1E-1H, Supplementary Table S5). The IC_50_ values revealed substantial intertumoral heterogeneity in response to all four drugs. Notably, more than half of the PDOs did not show clear dose-dependent cytotoxicity when treated with DTX or PTX, which further emphasized the heterogeneity in chemotherapy responses across different PDOs.

To investigate whether SIA-cIgG expression contributes to PDO variability in drug response, we quantified SIA-cIgG protein levels in the 7 PDOs (Figure 1I) and correlated them with their respective drug IC_50_ values. We found a marginally significant positive correlation between SIA-cIgG expression and CDDP IC_50_ (Figure 1J), with a Pearson correlation coefficient of *R* = 0.511, *P* = 0.071. In contrast, SIA-cIgG expression showed weak or no correlation with IC_50_ values for 5-FU, DTX, or PTX (Supplementary Figure S1A-S1C). These results suggest that high SIA-cIgG expression may contribute to primary CDDP resistance. Since current standard HNSCC chemotherapy regimens are predominantly CDDP-based, targeting SIA-cIgG could present a viable strategy for overcoming such primary resistance.

### Chemotherapy agents upregulate SIA-cIgG expression, and SIA-cIgG inhibition enhances drug efficacy

**Figure 2:**
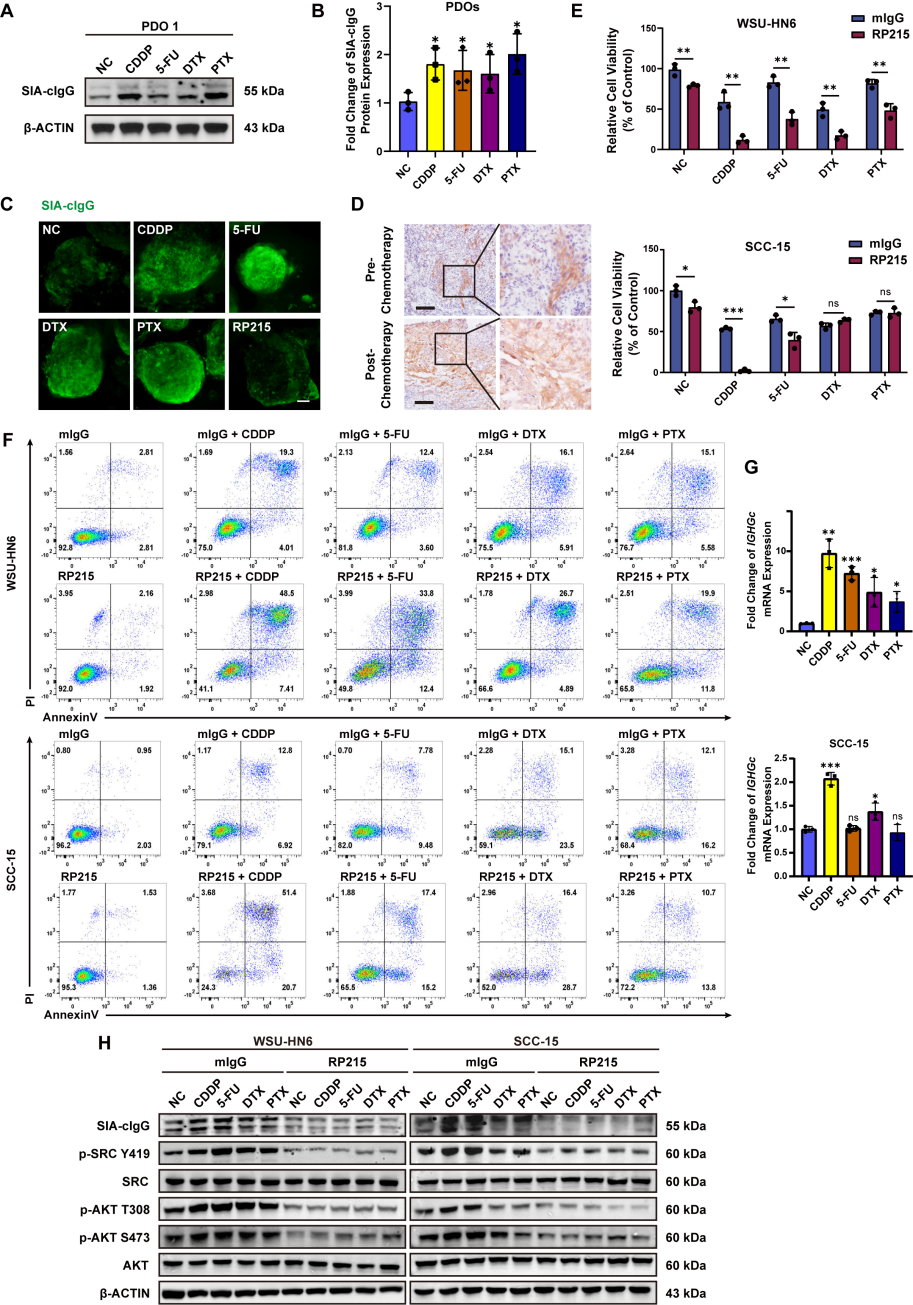
Chemotherapy agents upregulate SIA-cIgG expression, and SIA-cIgG inhibition enhances drug efficacy. (A) SIA-cIgG protein expression of PDO 1 after different drug treatments for 6 d (medium and drugs renewed in day 4) by Western blot assay. CDDP: 10 μmol/L; 5-FU: 12 μmol/L; DTX: 5 μmol/L; PTX: 5 μmol/L. (B) Fold change of SIA-cIgG protein expression after different drug treatments for 6 days in PDOs, *n* = 3 (respectively). (C) Representative SIA-cIgG fluorescence microphotographs of HNSCC PDOs after different drug treatments for 6 days (medium and drugs renewed in day 4). CDDP: 10 μmol/L; 5-FU: 12 μmol/L; DTX: 5 μmol/L; PTX: 5 μmol/L; RP215: 20 μg/mL. Scale bar, 50 μm. (D) Representative microphotographs of SIA-cIgG IHC staining in HNSCC tumor tissue, pre- (above) and post- (below) TPF (CDDP, 5-FU and DTX) chemotherapy. Scale bars, 100 μm. (E) Relative cell viability (% of control) of WSU-HN6 and SCC-15 after different drug combination treatments with/without 20 μg/mL RP215 for 48 h, *n* = 3 (respectively). 20 μg/mL mIgG was used as negative control to RP215. CDDP: 10 μmol/L; 5-FU: 12 μmol/L; DTX: 5 nmol/L; PTX: 5 nmol/L. (F) Flow cytometry analysis of AnnexinV/PI staining for indicating cell apoptosis after different drug combinations treatment for 48 h in WSU-HN6 and SCC-15, *n* = 3 (respectively). (G) Fold change of IGHGc mRNA expression after different drug treatments for 24 h in WSU-HN6 and SCC-15, *n* = 3 (respectively). (H) SIA-cIgG, p-SRC Y419, SRC, p-AKT T308, p-AKT S473, and AKT protein expression after different combination treatments for 36 h in WSU-HN6 and SCC-15, *n* = 3 (respectively). Data are represented as the mean ± SEM; ^*^P < 0.05, ^**^P < 0.01, ^***^P < 0.001, ns, no significant difference.

Patients frequently develop acquired resistance following chemotherapy.^[36]^ To determine whether chemotherap drugs affect SIA-cIgG expression, we treated PDOs with CDDP, 5-FU, DTX, or PTX. Consistent upregulation of SIA-cIgG was confirmed by Western blot (Figure 2A and Supplementary Figure S2A) and immunofluorescence (Figure 2C). Analysis of SIA-cIgG protein expression levels across 3 PDOs reveals that, despite the variations of response among PDOs, an overall increase in SIA-cIgG protein was consistently observed after drug treatment (Figure 2B). To further validate this observation in clinical samples, we analyzed paired pre- and post-chemotherapy tumor specimens from an HNSCC patient who received TPF regimen (CDDP, 5-FU, and DTX) and subsequently experienced disease progression. IHC revealed increased SIA-cIgG expression in the post-treatment tumor tissue (Figure 2D). This supports the hypothesis that chemotherapy-induced SIA-cIgG upregulation may contribute to the development of drug resistance and disease progression in HNSCC.

We then selected two HNSCC cell lines for further mechanistic studies: WSU-HN6 (low SIA-cIgG expression) and SCC-15 (high SIA-cIgG expression) (Supplementary Figure S2B, protein quantification in Supplementary Figure S2C) to test whether RP215 (a monoclonal antibody against SIA-cIgG) could neutralize this upregulation and increase chemosensitivity. Cells were pretreated with RP215 or control mouse IgG (mIgG), then exposed to the four drugs for 48 h. In WSU-HN6 cells, RP215 significantly enhanced the cytotoxicity of all four agents compared to the mIgG control. In SCC-15 cells, RP215 sensitized cells to CDDP and 5-FU but had little effect on responses to DTX or PTX (Figure 2E). Corresponding IC_50_ analyses confirmed these patterns (Supplementary Figure S2D-S2K). Apoptosis was assessed by Annexin V/PI staining and flow cytometry. In WSU-HN6 cells, RP215 increased apoptosis when combined with CDDP, 5-FU, DTX and PTX. In SCC-15 cells, enhanced apoptosis was seen with RP215 plus CDDP or 5-FU (Figure 2F). Together, these data indicate that blocking SIA-cIgG promotes chemotherapy-induced apoptosis.

Activation of the AKT signaling pathway is important for cell survival.^[37]^ Our prior work demonstrated that CDDP upregulates SIA-cIgG, leading to activation of the SRC/AKT pathway.^[29]^ In the present study, we found that CDDP, 5-FU, DTX, and PTX increased the mRNA level of the total IgG heavy chain (Figure 2G). These drugs also elevated SIA-cIgG protein levels and enhanced phosphorylation of both SRC and AKT in a dose-dependent manner (Supplementary Figure S2L, protein quantification in Supplementary Figure S2M). Treatment with RP215 reduced SIA-cIgG expression and suppressed the phosphorylation of SRC and AKT. Furthermore, RP215 blocked the drug-induced increase in SRC/AKT phosphorylation in both cell lines (Figure 2H, protein quantification in Supplementary Figure S2N). These results indicate that anti-SIA-cIgG therapy can enhance chemotherapy-induced cell death by inhibiting activation of the SRC/AKT signaling axis, irrespective of basal SIA-cIgG expression.

### Anti-SIA-cIgG inhibits HNSCC cell stemness and malignant biological behaviors

Chemoresistance is a primary manifestation of tumor stemness.^[36]^ Given that SIA-cIgG is highly expressed in non-small cell lung cancer stem cells and is crucial for maintaining stemness.^[26]^ We hypothesized that SIA-cIgG promotes chemoresistance in HNSCC by regulating tumor stemness.

**Figure 3:**
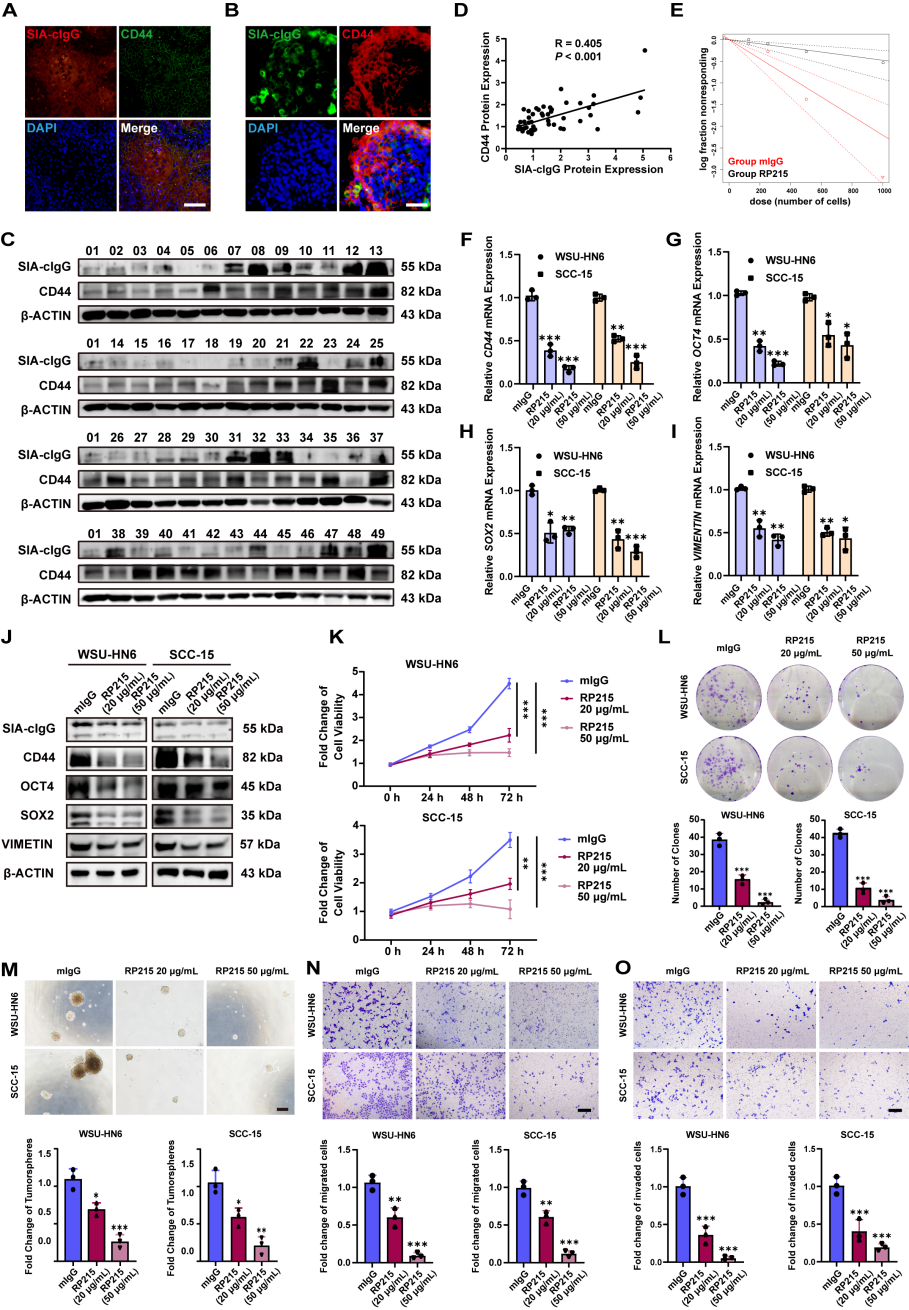
Anti-SIA-cIgG inhibits HNSCC cell stemness and malignant biological behaviors. (A) Representative fluorescence microphotographs of SIA-cIgG/CD44 staining in HNSCC tumor tissue. Scale bar, 100 μm. (B) Representative fluorescence microphotographs of SIA-cIgG/CD44 staining in HNSCC PDOs. Scale bar, 50 μm. (C) SIA-cIgG and CD44 protein expression of HNSCC tumor tissue by Western blot assay, *n* = 49. Sample 01 was used as control. (D) Simple linear regression of SIA-cIgG and CD44 protein expression, *n* = 49. (E) *In vitro* Extreme Limiting Dilution Analysis (ELDA) of 20 μg/mL mIgG or RP215-treated HNSCC PDOs. (F-I) CD44 (F), OCT4 (G), SOX2 (H) and VIMENTIN (I) mRNA expression after 20 μg/mL or 50 μg/mL RP215 treatment for 48 h, *n* = 3 (respectively). (J) SIA-cIgG, CD44, OCT4, SOX2, and VIMENTIN protein expression after 20 μg/mL or 50 μg/mL RP215 treatment for 48 h. (K) Cell proliferation rates of 20 μg/ mL or 50 μg/mL RP215 treatment in WSU-HN6 and SCC-15. 12 h after cell seeded was set as 0 h, *n* = 3 (respectively). (L-O) Colony formation assay (L), *in vitro* tumorsphere formation assay (M), Transwell migration (N) and invasion (O) of 20 μg/mL or 50 μg/mL RP215 treatment in WSU-HN6 and SCC-15, *n* = 3 (respectively). Scale bars, M: 250 μm; N-O: 200 μm. Data are represented as the mean ± SEM; ^*^P < 0.05, ^**^P < 0.01, ^**^P < 0.001, ns, no significant difference.

Immunofluorescence staining of HNSCC tissue revealed that SIA-cIgG-positive cell populations substantially overlapped with CD44-positive populations (Figure 3A), a known cancer stem cell marker, but not with those expressing the epithelial marker pan-cytokeratin (pan-CK) (Supplementary Figure S3A). A similar spatial overlap between SIA-cIgG and CD44 was observed in PDOs (Figure 3B). Analysis of 49 HNSCC tumor specimens (Figure 3C) revealed a positive correlation between SIA-cIgG and CD44 protein levels (*R* = 0.405, *P* < 0.001, Figure 3D). *In vitro* assessment of PDO stemness by Extreme Limiting Dilution Analysis (ELDA)^[38]^ showed that RP215 treatment reduced tumorsphere formation, indicating inhibition of stemness (Figure 3E).

The malignant biological behaviors of WSU-HN6 and SCC-15 also followed a trend consistent with their SIA-cIgG expression levels. Compared to proliferation (Supplementary Figure S3B) and colony formation (Supplementary Figure S3C), SCC-15 exhibited stronger capabilities in stemness-associated malignant behaviors such as sphere formation (Supplementary Figure S3D) and migration/ invasion (Supplementary Figure S3E), corresponding to its higher expression of stemness markers (Supplementary Figure S3F). Treatment of both cell lines with increasing concentrations of RP215 caused a dose-dependent downregulation of the stemness markers CD44, OCT4, and SOX2 at both mRNA (Figure 3F-3I) and protein levels (Figure 3J, protein quantification in Supplementary Figure S3G). Additionally, the epithelial-mesenchymal transition (EMT) marker VIMENTIN, which has been linked to stemness and chemoresistance,^[39,40]^ was also suppressed by RP215 treatment. RP215 also inhibited proliferation (Figure 3K), colony formation (Figure 3L), and tumorsphere formation (Figure 3M) in a dose-dependent manner. Transwell assays demonstrated that RP215 significantly reduced cell migration and invasion (Figure 3N-3O). Collectively, these findings indicate that SIA-cIgG is closely associated with tumor stemness and that inhibiting SIA-cIgG can suppress multiple malignant behaviors by targeting stemness.

### PTPN13 is a key downstream effector of SIA-cIgG in regulating tumor stemness and chemoresistance

Our prior work showed that PTPN13 is negatively regulated by SIA-cIgG and mediates SIA-cIgG-induced CDDP resistance by modulating SRC Y419 phosphorylation. However, the functional role of PTPN13 in HNSCC remains poorly understood. Given the association between SIA-cIgG and CD44, we hypothesized that PTPN13 might also function downstream of SIA-cIgG to regulate stemness.

**Figure 4:**
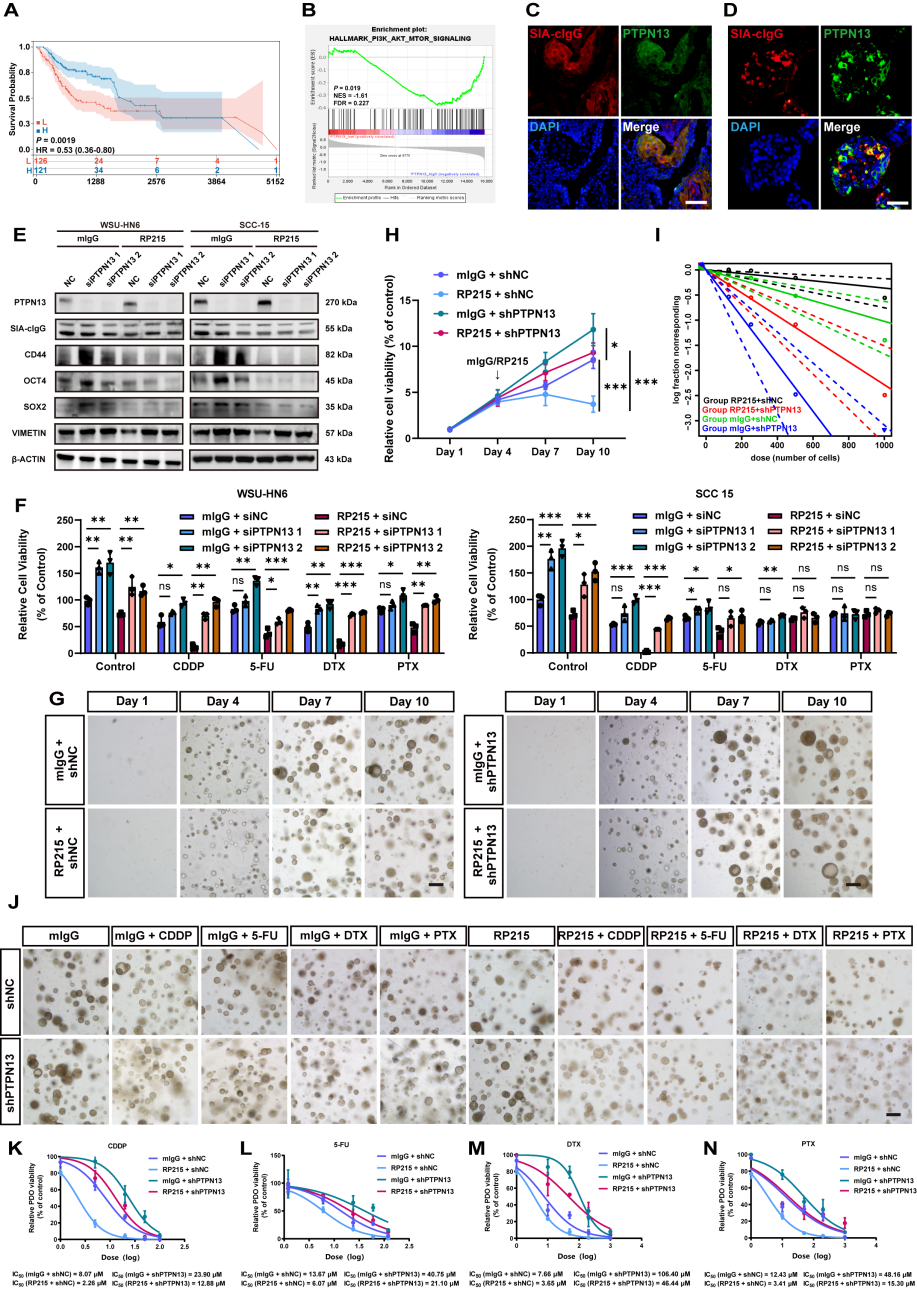
PTPN13 is a key downstream effector of SIA-cIgG in regulating tumor stemness and chemoresistance. (A) Kaplan-Meier survival analysis of HNSCC patients with high and low PTPN13 expression. High (H) = 124, Low (L) = 121. (B) GSEA Hallmark gene set enrichment analysis in PI3K/AKT pathway of TCGA comparing low versus high PTPN13 expression. (C) Representative fluorescence microphotographs of SIA-cIgG/PTPN13 co-localization in HNSCC tumor tissue. Scale bar, 100 μm. (D) Representative fluorescence microphotographs of SIA-cIgG/PTPN13 co-localization in HNSCC PDOs. Scale bar, 50 μm. (E) PTPN13, SIA-cIgG, CD44, OCT4, SOX2, and VIMENTIN protein expression of PTPN13 knockdown with/without 20 μg/mL RP215 treatment for 48 h. (F) Relative cell viability (% of control) of WSU-HN6 and SCC-15 after different drug treatments with/without PTPN13 knockdown as well as 20 μg/mL RP215 treatment for 48 h. SiNC was used as negative control to siPTPN13, 20 μg/mL mIgG was used as negative control to RP215, *n* = 3 (respectively). (G) Representative microphotographs of shNC or shPTPN13 HNSCC PDOs with/without 20 μg/mL RP215 treatment. Scale bar, 250 μm. (H) PDOs proliferation rates of shNC or shPTPN13 HNSCC PDOs with/without 20 μg/mL RP215 treatment. ^*^P < 0.05, ^***^P < 0.001. (I) *In vitro* Extreme Limiting Dilution Analysis (ELDA) of shNC or shPTPN13 HNSCC PDOs with/without 20 μg/mL RP215 treatment. (J) Representative microphotographs of shNC or shPTPN13 HNSCC PDOs after different drug treatments with/without 20 μg/mL RP215 treatment. CDDP: 10 μmol/L; 5-FU: 12 μmol/L; DTX: 5 μmol/L; PTX: 5 μmol/L. Scale bar, 250 μm. (K-N) Drug response curves of CDDP (K), 5-FU (L), DTX (M) and PTX(N) in shNC or shPTPN13 HNSCC PDOs with/without 20 μg/mL RP215 treatment. Data are represented as the mean ± SEM; ^*^P < 0.05, ^**^P < 0.01, ^***^P < 0.001, ns, no significant difference.

Analysis of the TCGA-HNSCC dataset indicated that low PTPN13 expression correlated with poorer prognosis (Figure 4A). GSEA Hallmark gene set enrichment analysis confirmed that high expression of PTPN13 was negatively associated with the PI3K/AKT pathway (Figure 4B). Immunofluorescence demonstrated co-localization of SIA-cIgG and PTPN13 in HNSCC tissues and PDOs (Figure 4C-4D, quantitative analysis in Supplementary Figure S4A). To evaluate the functional role of PTPN13, we knocked down its expression using two independent siRNAs and assessed stemness markers with or without RP215 treatment. Knockdown of PTPN13 with two independent siRNAs increased the mRNA (Supplementary Figure S4B-S4E) and protein levels of CD44, OCT4, SOX2, and VIMENTIN. It also reversed the RP215-mediated suppression of VIMENTIN, though not that of the stemness markers (Figure 4E; protein quantification in Supplementary Figure S4F). Meanwhile, PTPN13 knockdown promoted cell proliferation, migration, and invasion, and completely abolished the inhibitory effects of RP215 on these malignant behaviors (Supplementary Figure S5A-S5B). A stable PTPN13 knockdown (shPTPN13) cell line was established (knockdown efficiency shown in Supplementary Figure S5C), which completely reversed the RP215-induced suppression of colony and sphere formation (Supplementary Figure S5D-S5E). Crucially, PTPN13 knockdown abolished the RP215-induced sensitization to CDDP, 5-FU, DTX, and PTX in WSU-HN6 cells, and to CDDP and 5-FU in SCC-15 cells (Figure 4F). Western blot analysis revealed that shPTPN13 reversed the inhibitory effect of RP215 on the drug-induced elevation of SRC/AKT phosphorylation levels (Supplementary Figure S5F, protein quantification in Supplementary Figure S5G). Stable PTPN13 knockdown in PDOs (knockdown efficiency shown in Supplementary Figure S6A) completely reversed the inhibitory effects of RP215 on PDO growth rate (Figure 4G-4H) and further enhanced with passaging (Fig. S6B-S6D). ELDA confirmed that PTPN13 knockdown reversed the RP215-induced suppression of tumorsphere formation (Figure 4I). In PDOs, PTPN13 knockdown reduced drug cytotoxicity and rescued the chemosensitizing effect of RP215 (Figure 4J) to CDDP (Figure 4K), 5-FU (Figure 4L), DTX (Figure 4M), and PTX (Figure 4N).

In summary, these data identify PTPN13 as a critical downstream effector of SIA-cIgG in regulating tumor stemness and further driving chemoresistance in HNSCC.

### Anti-SIA-cIgG enhances PTPN13 protein stability

**Figure 5:**
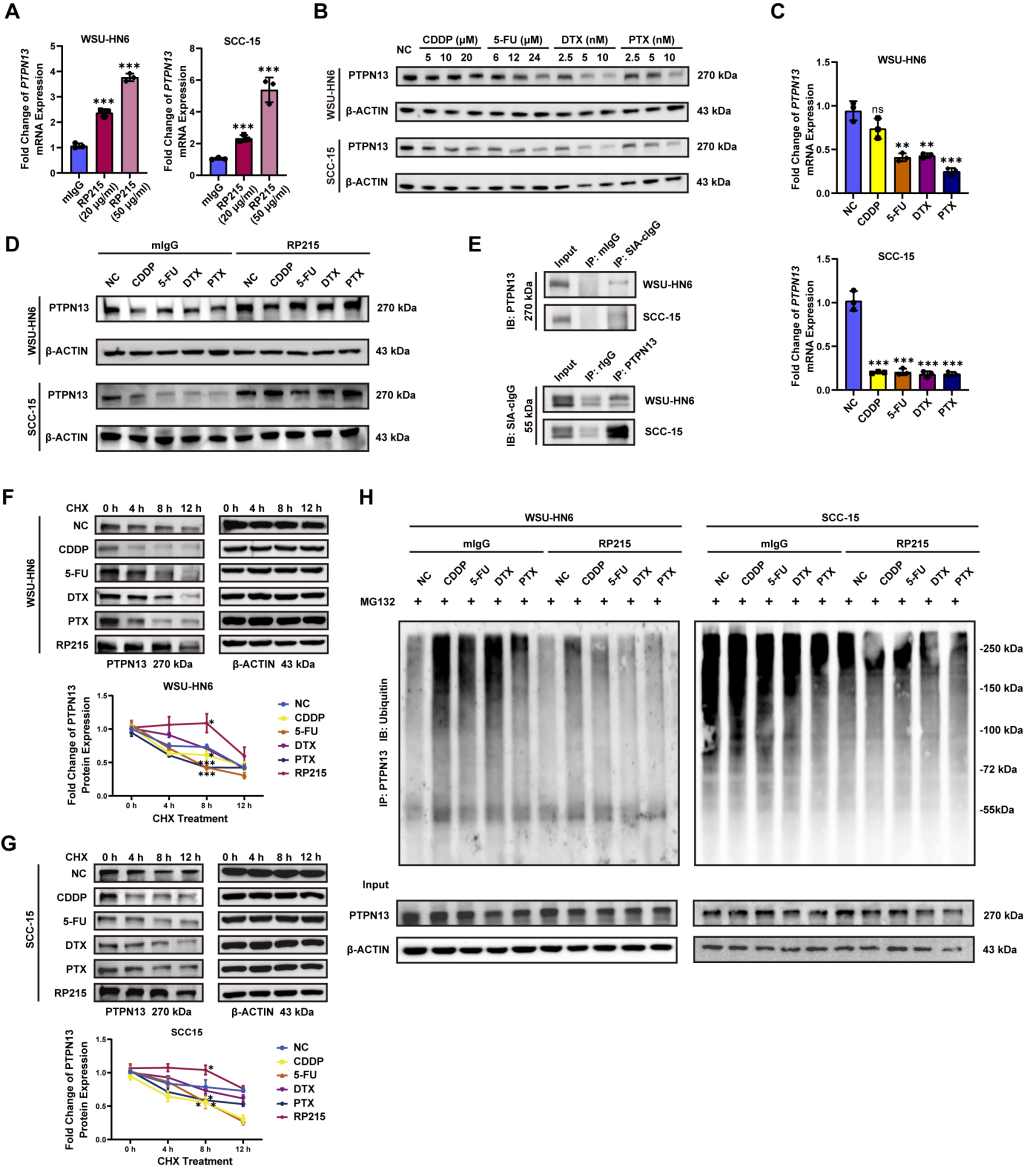
Anti-SIA-cIgG enhances PTPN13 protein stability. (A) Fold change of PTPN13 mRNA expression after 20 μg/mL or 50 μg/mL of RP215 treatment for 24 h in WSU-HN6 and SCC-15, *n* = 3 (respectively). (B) PTPN13 protein expression after treatments of different drugs at different concentration for 48 h in WSU-HN6 and SCC-15. (C) Fold change of PTPN13 mRNA expression after different drugs treatment for 24 h in WSU-HN6 and SCC-15, *n* = 3 (respectively). (D) PTPN13 protein expression of WSU-HN6 and SCC-15 after different drugs treatment with/without 20 μg/mL RP215 for 48 h. (E) Binding of PTPN13 to SIA-cIgG in WSU-HN6 and SCC-15 determined by IP. (F, G) CHX chase assay for indicating PTPN13 protein half-life time in WSU-HN6 (F) and SCC-15 (G). After different drugs treatment for 24 h, 50 μg/mL CHX was added into WSU-HN6 or SCC-15 cells for 0, 4, 8, 12 h, Western blot assay was performed to observe PTPN13 protein expression. (H) Ubiquitin protein expression of PTPN13 in WSU-HN6 after different drugs treatment with/without 20 μg/mL RP215 for 24 h, followed by MG132 treatment for 6 h. MG132: 10 μmol/L. Data are represented as the mean ± SEM; ^*^P < 0.05, ^**^P < 0.01, ^***^P < 0.001, ns, no significant difference.

Treatment of WSU-HN6 and SCC-15 cells with increasing concentrations of RP215 led to elevated PTPN13 mRNA levels (Figure 5A). Unexpectedly, although all four chemotherapeutic drugs caused a dose-dependent reduction of PTPN13 protein in both cell lines (Figure 5B, protein quantification in Supplementary Figure S7A) and decreased PTPN13 mRNA in SCC-15 cells, CDDP treatment did not significantly alter PTPN13 mRNA expression in WSU-HN6 cells (Figure 5C). Nevertheless, RP215 effectively restored PTPN13 protein expression and reversed drug-induced PTPN13 downregulation (Figure 5D, protein quantification in Supplementary Figure S7B).

To examine a potential interaction between SIA-cIgG and PTPN13, we performed co-immunoprecipitation (Co-IP) assays. PTPN13 was detected in the SIA-cIgG immunoprecipitation, and conversely, SIA-cIgG was found in the PTPN13 immunoprecipitation (Figure 5E), suggesting a potential interaction.

We next assessed the effects of chemotherapy and RP215 on PTPN13 protein stability. Cells were treated with CDDP, 5-FU, DTX, PTX, or RP215, followed by the protein synthesis inhibitor cycloheximide (CHX). Western blot analysis at 0, 4, 8, and 12 h post-CHX showed that CDDP, 5-FU, and PTX significantly shortened the half-life of PTPN13, whereas DTX had little effect. In contrast, RP215 treatment stabilized PTPN13 and prolonged its half-life (Figure 5F-5G). Control experiments with actinomycin D (ActD) excluded RNA stability effects (Supplementary Figure S7C). Further analysis of PTPN13 ubiquitination levels after blocking degradation with MG 132 showed that the four drugs increased PTPN13 ubiquitination, while RP215 decreased it (Figure 5H). These findings collectively demonstrate that SIA-cIgG promotes PTPN13 protein degradation *via* ubiquitination, and that inhibiting SIA-cIgG stabilizes PTPN13 by reducing this modification.

### Anti-SIA-cIgG upregulates SP1 expression to activate PTPN13 promoter activity

**Figure 6:**
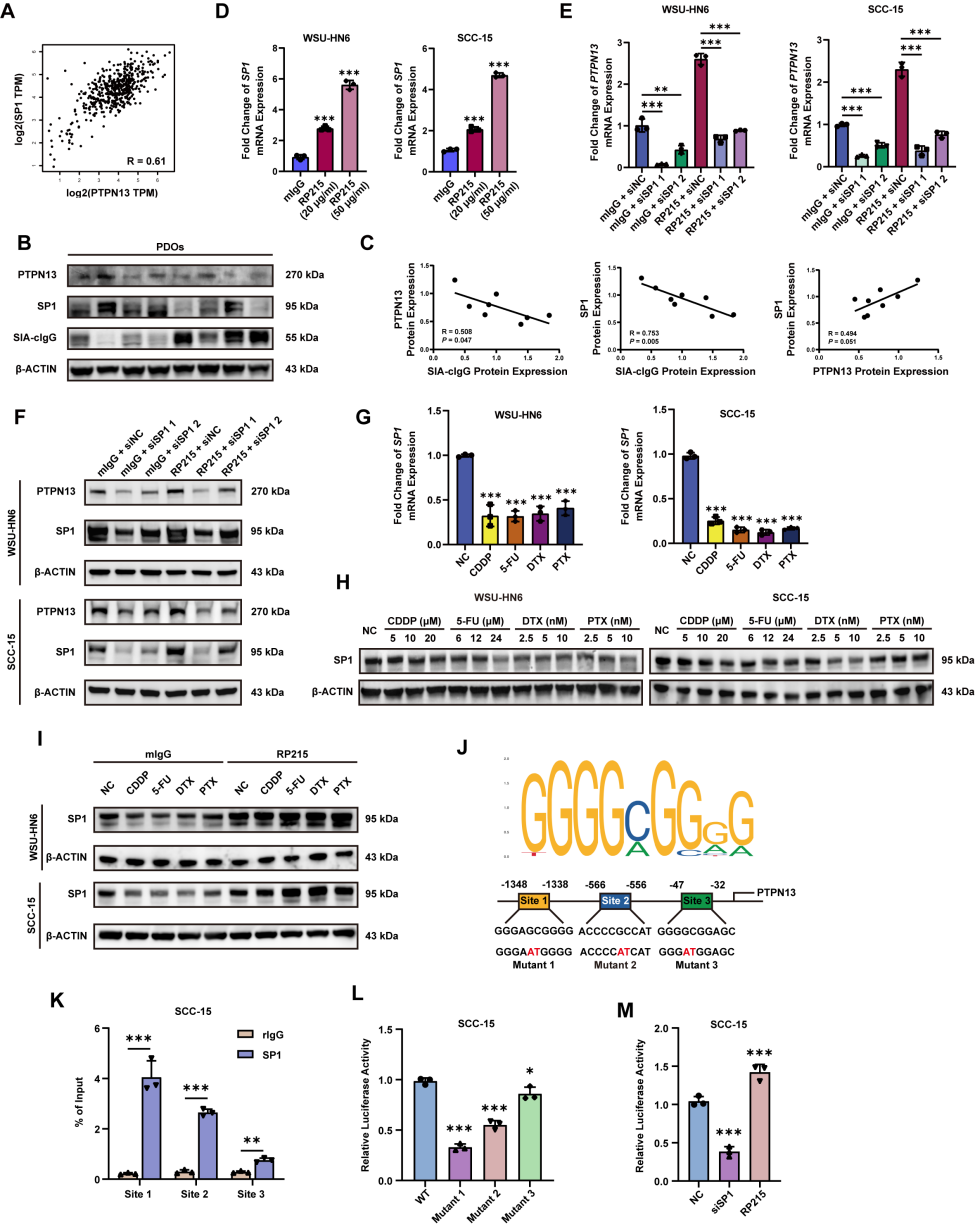
Anti-SIA-cIgG upregulates SP1 expression to activate PTPN13 promoter activity. (A) Correlation analysis from TCGA-HNSCC database showed the transcriptional relevant of PTPN13 and SP1 (Created with GEPIA: http://gepia.cancer-pku.cn/index.html). (B) SIA-cIgG, PTPN13 and SP1 protein expression of 8 different PDOs by Western blot assay. (C) Simple linear regression of SIA-cIgG protein expression and PTPN13 protein expression (left), SIA-cIgG protein expression and SP1 protein expression (middle), PTPN13 protein expression and SP1 protein expression (right), *n* = 8. (D) Fold change of SP1 mRNA expression after 20 μg/mL or 50 μg/mL RP215 treatment for 24 h in WSU-HN6 and SCC-15, *n* = 3 (respectively). (E) Fold change of PTPN13 mRNA expression after SP1 knockdown with/without 20 μg/mL RP215 for 24 h in WSU-HN6 and SCC-15, *n* = 3 (respectively). (F) PTPN13 and SP1 protein expression after SP1 knockdown with/without 20 μg/mL RP215 for 48 h in WSU-HN6 and SCC-15. (G) Fold change of SP1 mRNA expression after different drug treatment for 24 h in WSU-HN6 and SCC-15, *n* = 3 (respectively). (H) SP1 protein expression after treatments of different drugs at different concentration for 48 h in WSU-HN6 and SCC-15. (I) SP1 protein expression of WSU-HN6 and SCC-15 after different drugs treatment with/without 20 μg/mL RP215 for 48 h. (J) Three predicted SP1 binding site on PTPN13 promoter by JASPAR (jaspar.genereg.net). (K) Binding efficiency of SP1 to different sites in SCC-15. ChIP-qPCR assay was performed, and data are shown as the ratio of immunoprecipitated DNA to total input DNA (%), *n* = 3 (respectively). (L) Relative luciferase activity in SCC-15 transfected with pGL3-Enhancer-PTPN13-promoter-WT or pGL3-Enhancer-PTPN13-promoter-Mutant 1 or pGL3-Enhancer-PTPN13-promoter-Mutant 2 or pGL3-Enhancer-PTPN13-promoter-Mutant 3, as well as pRL-TK, *n* = 3. (M) Relative luciferase activity in SCC-15 transfected with pGL3-Enhancer-PTPN13-promoter-WT, siSP1/siNC, and treated with/without 20 μg/mL mIgG, *n* = 3. Data are represented as the mean ± SEM; ^*^P < 0.05, ^**^P < 0.01, ^***^P < 0.001, ns, no significant difference.

The ability of RP215 to upregulate PTPN13 mRNA suggested that SIA-cIgG also regulates PTPN13 at the transcriptional level. Bioinformatics screening of the JASPAR and UCSC databases for potential PTPN13 regulators identified SP1 as a promising candidate. Analysis of the TCGA-HNSCC dataset revealed a positive correlation between SP1 and PTPN13 mRNA expression (Figure 6A). To validate this finding, we measured SIA-cIgG, SP1, and PTPN13 protein levels in eight different PDOs. Consistent with our proposed axis, PDOs exhibiting higher SIA-cIgG expression consistently showed lower levels of both PTPN13 and SP1 proteins (Figure 6B). Quantitative correlation analysis confirmed a significant negative linear relationship between SIA-cIgG and PTPN13 (Figure 6C, left, *R* = 0.508, *P* = 0.047), and between SIA-cIgG and SP1 (Figure 6C, middle, *R* = 0.753, *P* = 0.005). In contrast, PTPN13 and SP1 protein levels showed a positive correlation (Figure 6C, right, *R* = 0.494, *P* = 0.051). To validate whether SP1 mediates SIA-cIgG-regulated PTPN13 transcription, we examined SP1 expression after RP215 or drug treatment. RP215 upregulated SP1 at both mRNA and protein levels. Conversely, SP1 knockdown (knockdown efficiency shown in Supplementary Figure S5A) abolished RP215-induced upregulation of PTPN13 mRNA (Figure 6E) and protein level (Figure 6F, protein quantification in Supplementary Figure S8B). In contrast, all 4 drugs led to dose-dependent downregulation of SP1 in both mRNA and protein level (Figure 6G-6H, protein quantification in Supplementary Figure S8C), and this downregulation was reversed by RP215 (Figure 6I, protein quantification in Supplementary Figure S8D).

To further elucidate the mechanism of SP1-mediated PTPN13 regulation, we analyzed the PTPN13 promoter region (–1500 to +100) using the JASPAR database and identified three potential SP1 binding sites (Fig. 6J). Chromatin immunoprecipitation (ChIP) assays in SCC-15 cells confirmed SP1 binding to all three sites, with site 1 showing the strongest enrichment (Figure 6K). Reporter plasmids containing wild-type (WT) or site-specific mutant PTPN13 promoters were constructed in the pGL3-Enhancer luciferase vector. Dual-luciferase assays revealed that mutation of site 1 caused a significant reduction in promoter activity, indicating its critical role in SP1-mediated transcriptional activation (Figure 6L). Furthermore, SP1 knockdown suppressed, whereas RP215 treatment enhanced, the luciferase activity driven by the PTPN13 promoter (Figure 6M).

These findings collectively demonstrate that SP1 binds directly to the PTPN13 promoter and activates its transcription. SIA-cIgG downregulates PTPN13 by suppressing SP1 expression, whereas anti-SIA-cIgG (RP215) upregulates SP1 to restore PTPN13 transcription.

### Stable knockdown of PTPN13 in xenograft tumors reversed the therapeutic benefits of anti-SIA-cIgG combination therapy

**Figure 7:**
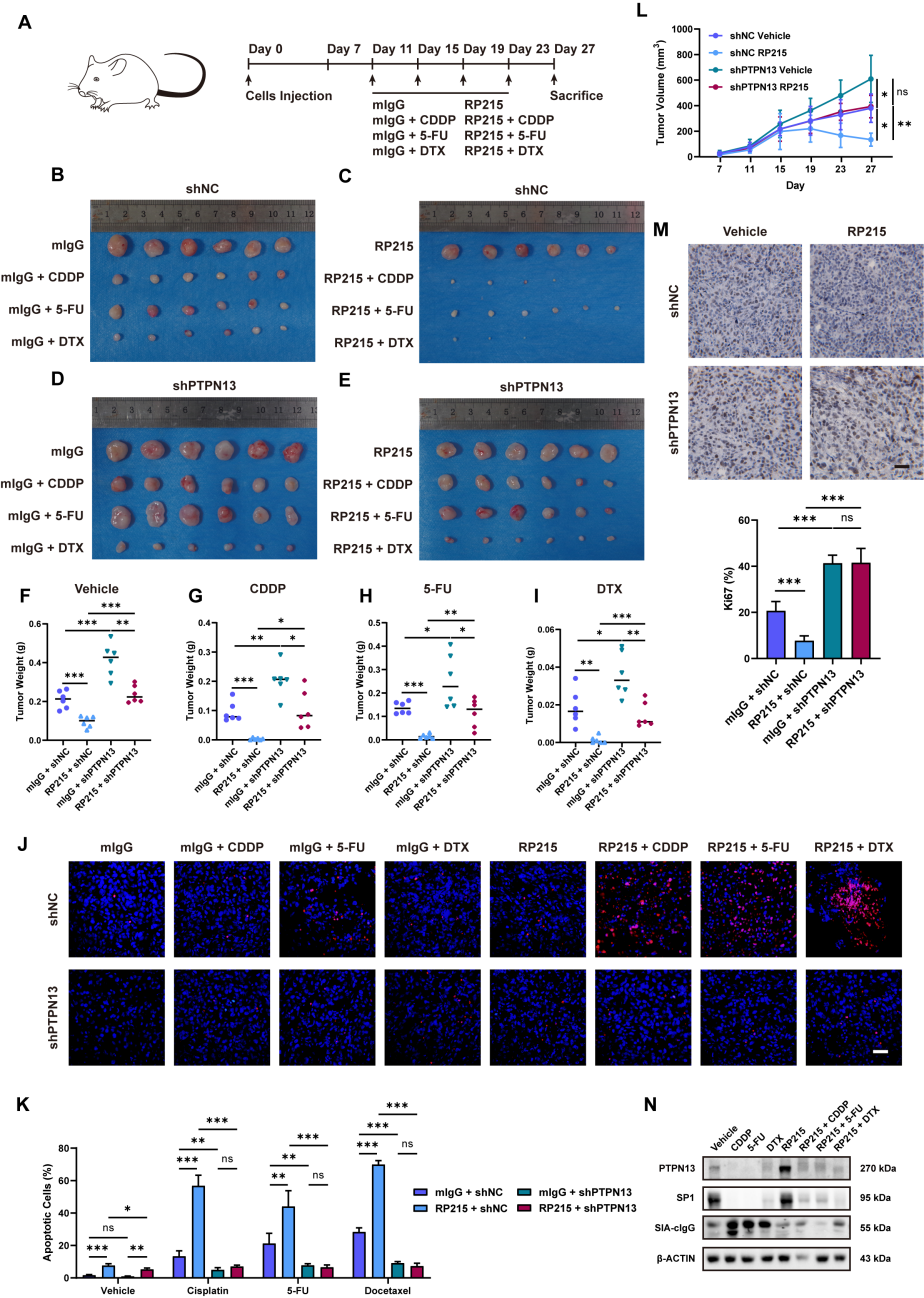
Stable knockdown of PTPN13 in xenograft tumors reversed the therapeutic benefits of anti-SIA-cIgG combination therapy. (A) Schematic timeline of *in vivo* experiment. (B-E) Representative images of tumor volumes of different drug treatments on day 27, CDDP: 2 mg/kg; 5-FU: 20 mg/kg; DTX: 2 mg/kg; mIgG/RP215: 2 mg/kg. (F-I) Tumor weight of different drug treatments and PTPN13 condition on day 27. (J) TUNEL staining of xenograft tumor tissues with different drug treatments and PTPN13 condition. Scale bars, 100 μm. (K) Quantification analysis of TUNEL staining. (L) Tumor growth kinetics of shNC or shPTPN13 HNSCC PDOs with/without RP215 treatment. (M) Representative microphotographs and quantification analysis of Ki67 IHC staining in xenograft tumor tissues. Scale bars, 100 μm. (N) PTPN13, SP1 and SIA-cIgG protein expression in vehicle, CDDP, 5-FU and DTX treatment with/without RP215. Data are represented as the mean ± SEM; ^*^P < 0.05, ^**^P < 0.01, ^***^P < 0.001, ns, no significant difference.

To evaluate the therapeutic potential and biosafety of RP215 combined with chemotherapy and to define the role of PTPN13 in SIA-cIgG-mediated chemoresistance, we established xenograft models using WSU-HN6 shNC and shPTPN13 cells. Treatment started when tumors reached approximately 100 mm^3^ (Schematic timeline: Figure 7A).

After four treatment cycles, RP215-based combinations showed significant antitumor efficacy (Figure 7B-7C, H&E staining in Supplementary Figure S9A). Complete tumor regression was observed 2/6 in the RP215 + CDDP group and 3/6 in the RP215 + DTX group. Compared to shNC controls, shPTPN13 xenografts exhibited substantially diminished treatment response (Figure 7D-7E, H&E staining in Supplementary Figure S9A). Although RP215 combinations remained more effective than chemotherapy alone in shPTPN13 models, the degree of therapeutic enhancement was markedly reduced (Figure 7F-7I). TUNEL staining (Figure 7J-7K) results confirmed that the combination of RP215 with drugs significantly increased tumor cell apoptosis compared to single drug, and this proapoptotic effect was abolished by shPTPN13. ShPTPN13 xenografts also displayed accelerated tumor growth (Figure 7L), and Ki67 staining indicated that PTPN13 knockdown promoted tumor cell proliferation (Figure 7M).

Western blot analysis of xenograft tissues verified chemotherapy-induced upregulation of SIA-cIgG with concurrent downregulation of PTPN13 and its transcriptional regulator SP1 (Figure 7N, protein quantification in Supplementary Figure S9B). Comprehensive histopathological examination (H&E staining) of major organs (heart, liver, spleen, lung, kidney; Supplementary Figure S9C) and stable body weight monitoring (Supplementary Figure S9D) showed no significant treatment-related toxicity, supporting the favorable biosafety profile of anti-SIA-cIgG-based drug combinations.

### Anti-SIA-cIgG-based combination therapy exhibit significantly higher anti-cancer effect compared to existing chemotherapy regimens in HNSCC PDOs

**Figure 8:**
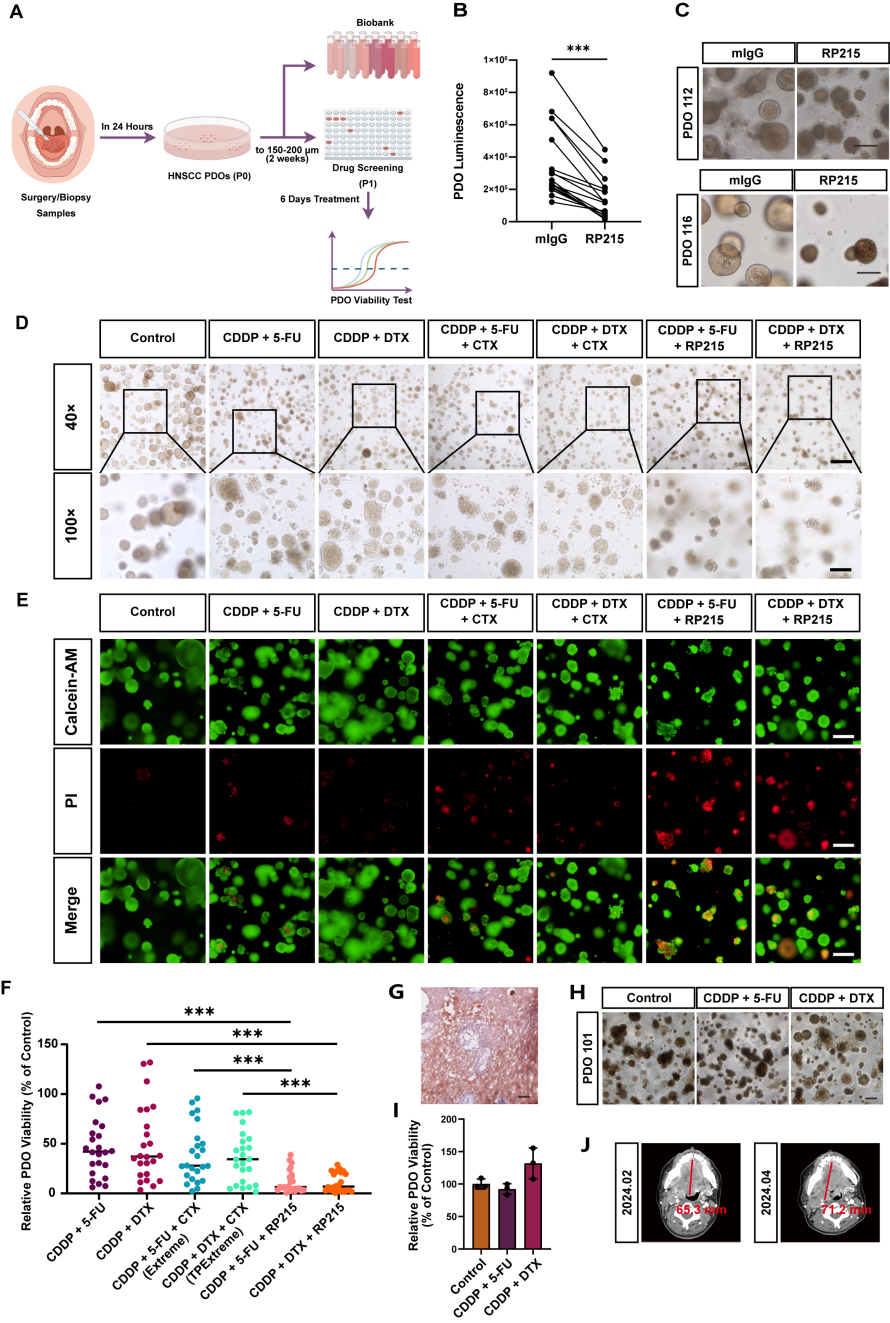
Anti-SIA-cIgG drug combinations show significantly higher anti-tumor effect than existing chemotherapy combinations in HNSCC PDOs. (A) Workflow of HNSCC PDOs establishment and drug screening (Generated by Figdraw). (B) Luminescence of mIgG or RP215-treated HNSCC PDOs for indicating cell viability, *n* = 17. (C) Representative microphotographs of 20 μg/mL mIgG or RP215-treated HNSCC PDOs. Scale bars, 100 μm. (D) Representative microphotographs of HNSCC PDOs after different drug combination treatments. Scale bars, 250 μm (above), 100 μm (below). CDDP: 10 μmol/L; 5-FU: 12 μmol/L; DTX: 5 μmol/L; PTX: 5 μmol/L; CTX: 100 μg/mL; RP215: 20 μg/mL. (E) Representative fluorescence microphotographs of Calcein-AM/PI staining for indicating cell viability/ cytotoxicity after different drug combination treatments. Scale bars, 100 μm. (F) Relative PDO viability (% of control) of HNSCC PDOs after different drug combination treatments, *n* = 25. (G) Representative microphotographs of SIA-cIgG IHC staining in patient 101 tumor tissue, Scale bar, 50 μm. (H) Representative microphotographs of PDO 101 after different treatments. Scale bar, 250 μm. (I) Relative PDO viability (% of control) of PDO 101 after different drug combination treatments. (J) Pre- and post-TPF chemotherapy CT images of patient 101. Data are represented as the mean ± SEM; ^***^P < 0.001. ns: no significant difference.

To more accurately model clinical drug responses and assess the translational potential of anti-SIA-cIgG therapy, we established first passage (P1) PDOs from HNSCC samples to evaluate the effects of different treatments on viability (workflow in Figure 8A). Treatment with RP215 (20 μg/mL) to neutralize SIA-cIgG (Supplementary Figure S7A) in 17 HNSCC PDOs significantly inhibited organoid growth and reduced PDO viability compared to untreated control group (Figure 8B; Supplementary Table S6). RP215-treated PDOs exhibited two distinct morphological patterns: in some cases (*e.g*., PDO 112), PDOs showed reduced diameters, decreased translucency, and irregular edges, suggesting surface cell death (Figure 8C, upper); in others (*e.g*., PDO 116), only size reduction was observed, with or without loss of translucency (Figure 8C, lower).

To compare the anticancer efficacy of anti-SIA-cIgG-based combinations with existing chemotherapy regimens, 25 PDOs were treated with two anti-SIA-cIgG-based drug combinations (CDDP + 5-FU + RP215 and CDDP + DTX + RP215) and four current clinical chemotherapy regimens: two dual-drug combinations (CDDP + 5-FU and CDDP + DTX) and 2 cetuximab-based drug combinations (CDDP + 5-FU + CTX [Extreme regimen] and CDDP + DTX + CTX [TPExtreme regimen]). Micrographs (Figure 8D) showed that untreated PDOs were large, with smooth edges and good translucency. PDOs treated with dual-drug or cetuximab-based drug combinations showed reduced size and partial edge disintegration. Notably, PDOs in the anti-SIA-cIgG-based combination therapy showed the smallest diameters, extensive disintegration, and poor translucency. Calcein-AM/PI staining further confirmed increased cell death in the anti-SIA-cIgG-based drug combinations (Figure 8E). Quantitative analysis (Figure 8F; Supplementary Table S7) demonstrated that although PDOs derived from different patients showed varied sensitivity to chemotherapy, the two anti-SIA-cIgG-based combination therapy consistently exhibited stronger tumor suppression than the dual-drug combinations or cetuximab-based drug combinations. Importantly, these combinations significantly overcame inter-patient heterogeneity in drug response.

To verify the consistency of PDOs drug responses and clinical patient chemotherapy response, we collected contrast-enhanced CT images from 9 patients who had corresponding PDOs established and underwent neoadjuvant chemotherapy with the TPF regimen (TPF regimen: CDDP + 5-FU + DTX) before and after treatment. The PDO results were compared with the actual clinical outcomes. Referring to previous report,^[41]^ a PDO viability rate below 70% (relative to control) was defined as an effective drug response, and a rate above 70% as ineffective. For clinical efficacy, a complete or partial response was considered effective, while stable disease or progression was considered ineffective. The results for 6 out of 9 PDOs were concordant with the clinical outcomes, yielding a consistency rate of 66.67% (Table 1).

**Table 1:** Consistency of PDOs drug responses and clinical patient chemotherapy responses

	PDOs viability (% relative to control)		Clinical chemotherapy	
No.	CDDP + 5-FU	CDDP + DTX	response	Consistency
089	39.41	48.33	Stable	Inconsistent
094	26.97	20.29	Partial response	Consistent
098	57.76	87.30	Progressing	Consistent
099	30.60	35.16	Partial response	Consistent
100	94.72	130.41	Partial response	Inconsistent
101	92.27	131.81	Progressing	Consistent
107	107.73	112.85	Stable	Consistent
115	12.40	37.60	Stable	Inconsistent
116	41.46	50.20	Partial response	Consistent

Furthermore, as shown in Figure 1H, patients with high SIA-cIgG expression (*e.g*., PDO 089, 098, 101) exhibited poor clinical responses (stable/progressing), whereas those with low SIA-cIgG expression (*e.g*., PDO 094, 099, 100) responded well (partial response). For instance, PDO 101, with high SIA-cIgG expression in both the tumor tissue (Figure 8G) and PDOs, showed minimal reduction in PDOs size (Figure 8H) and viability (Figure 8I) upon treatment with the dual-drug combinations, and no tumor shrinkage was observed in pre- *vs*. post-TPF CT images (Figure 8J). In contrast, PDO 099 with low SIA-cIgG expression showed reduced organoid size and viability after treatment, accompanied by partial tumor regression on CT (Supplementary Figure S7B-S7E).

Collectively, these results demonstrate that patients with high SIA-cIgG expression derive limited benefit from conventional chemotherapy. In contrast, anti-SIA-cIgG-based combination therapy exhibit superior anti-tumor efficacy across PDOs from various patients, highlighting their promising therapeutic potential in HNSCC treatment.

## Discussion

This study is the first to demonstrate the association between SIA-cIgG and resistance mechanisms to four commonly used chemotherapeutic drugs in HNSCC: CDDP, 5-FU, DTX, and PTX. High expression of SIA-cIgG contributes to primary resistance to CDDP and poorer clinical chemotherapy outcomes. Moreover, these drugs induce SIA-cIgG upregulation, which subsequently activates SRC/AKT pathway to promote cell survival and chemoresistance. Drug resistance, particularly multidrug resistance, is a primary manifestation of tumor stemness. ^[36]^ This study highlights the crucial role of SIA-cIgG and its downstream effector PTPN13 in regulating tumor stemness. Anti-SIA-cIgG therapy enhances PTPN13 protein stability and promotes its transcription *via* upregulation of the transcription factor SP1, thereby suppressing multiple malignant behaviors and improving chemosensitivity. Most importantly, this study is the first to demonstrate the significant therapeutic efficacy of anti-SIA-cIgG-based drug combinations over current clinical regimens in HNSCC PDOs, providing strong preclinical support for their potential clinical translation.

A major factor limiting chemotherapy efficacy in HNSCC is the lack of effective therapeutic targets.^[4]^ Although over 90% of HNSCC cases exhibit EGFR overexpression, and cetuximab-based drug combinations are widely used in clinical practice, reported therapeutic outcomes have shown contradictory results.^[42]^ SIA-cIgG is broadly expressed in epithelial malignancies and may serve as a predictive biomarker.^[13,16,17,18,19,21]^ Previous studies indicate that SIA-cIgG promotes tumor progression through multiple mechanisms: mediating immune evasion by binding T‑cell Siglecs,^[15]^ enhancing migration and invasion *via* the integrin β4/FAK/SRC pathway,^[17]^ and maintaining cancer stemness through a SIA-cIgG/c‑MET/SOX2 autoregulatory loop.^[26]^ In pancreatic cancer, SIA-cIgG positivity correlates with poor pathological response to neoadjuvant chemotherapy,^[25]^ yet its functional role in chemotherapy response had not been explored. Our findings demonstrate that SIA-cIgG expression levels influence tumor chemosensitivity. In PDOs, higher SIA-cIgG expression showed a linear correlation with a higher CDDP IC_50_, suggesting a role in primary CDDP resistance. Since most chemotherapeutic regimens are CDDP-based, we examined patients’ chemotherapy outcomes and found that PDOs with high SIA-cIgG expression showed minimal viability reduction after drug treatment, and the corresponding patients experienced no tumor remission. Conversely, PDOs with low SIA-cIgG expression were drug-sensitive, and their corresponding patients showed partial tumor remission. These observations support SIA-cIgG as a potential predictive biomarker for chemotherapy response and indicate the possible clinical benefit of anti-SIA-cIgG combination therapy.

AKT activation is a key mechanism underlying multidrug resistance,^[43]^ and its phosphorylation indicates activation. Our previous work^[25]^ showed that CDDP upregulates SIA-cIgG, which then suppresses the phosphatase PTPN13, leading to increased phosphorylation of SRC and AKT, particularly at T308, a site critical for AKT activation, proliferation, and survival.^[44]^ In the present study, CDDP and 5-FU robustly increased phosphorylation of both SRC (Y419) and AKT (T308/S473) in WSU-HN6 and SCC-15 cells. In contrast, DTX and PTX increased SRC phosphorylation but failed to activate AKT in SCC-15 cells. This result also corresponded with the result of apoptosis: RP215 enhanced apoptosis when combined with CDDP or 5-FU but showed little effect when combined with DTX or PTX in SCC-15 cells. Previous studies indicate that active SRC is translocated into lipid rafts *via* scaffold proteins to activate specific downstream pathways, and lipid raft homeostasis is critical for SRCmediated PI3K/AKT activation.^[45,46]^ We therefore infer that RP215 promotes drug‑induced apoptosis primarily by blocking productive SRC/AKT activation, and that cell‑specific differences in drug response may relate to drug‑induced alterations in lipid‑raft homeostasis. This concept of context‑dependent signaling is further supported by our PDO data, where roughly half of the PDOs were more sensitive to the CDDP + 5‑FU + RP215 combination and the other half to CDDP + DTX + RP215, with no single regimen being universally superior. Future studies investigating how genetic background influences SRC/AKT activation will be valuable.

Cancer stem cells (CSCs) drive chemoresistance through their self‑renewal and tumor‑initiating capacity.^[36]^ In this study, we showed SIA-cIgG/PTPN13 axis plays an important role in cancer stemness. With our observation that chemotherapy upregulates SIA-cIgG and that SIA-cIgG expression correlates with drug IC_50_, we propose that treatment preferentially eliminates less stem‑like cells, enriching for high‑SIA‑cIgG populations with stronger stemness, thereby increasing post‑therapy SIA‑cIgG levels. PTPN 13 is a key downstream effector in this axis. PTPN13 knockdown promotes stemness and chemoresistance and completely reverses the RP215‑mediated suppression of malignant behaviors. Although PTPN13 knockdown reversed RP215-induced downregulation of VIMENTIN, it did not restore the expression of the stemness markers CD44, OCT4, or SOX2. Previous studies have demonstrated that the stemnessrelated transcription factors SOX2 and OCT4 bind to the promoter of SIAcIgG and enhance its expression. In turn, SIAcIgG can interact with cMET and CD44, activating downstream PI3K/AKT and RAS/ERK pathways, and forming a selfpropagating cMet/SOX2/SIAcIgG loop.^[26]^ As a downstream effector of SIAcIgG that modulates SRC/AKT pathway, PTPN13 may also regulate the expression of SOX2 and OCT4 through its influence on AKT phosphorylation levels.^[47,48]^ However, since SIAcIgG can still regulate SOX2 through RAS/ERK pathway, knockdown of PTPN13 alone is insufficient to reverse RP215‑mediated suppression of these transcription factors. In contrast, VIMENTIN, a key effector protein in the EMT process of tumor cells, is regulated differently. Knockdown of PTPN13 upregulates the expression of four EMTdriving transcription factors: SLUG, SNAIL, ZEB1, and ZEB2,^[32]^ thereby completely reversing the RP215mediated inhibition of VIMENTIN. Together, these findings suggest that SIAcIgG likely participates in tumor stemness through multiple parallel pathways. Based on our data, the SIAcIgG/PTPN13 axis appears to play a more prominent role in mediating chemotherapy resistance and EMT, rather than in the direct regulation of core stemness transcription factors.

SP1 is a well-known transcription factor with complex functions regulated by post-translational modifications.^[49]^ Our observation suggests that SIA-cIgG negatively regulates the SP1/PTPN13 axis. Anti-SIA-cIgG treatment increased PTPN13 protein stability and enhanced its transcription *via* SP 1-mediated promoter activation. While this study did not include SIA-cIgG gain-of-function experiments, the reversal of anti-SIA-cIgG effects by PTPN13 knockdown confirms the critical role of PTPN13 as a downstream effector of SIA-cIgG. However, when further exploring the function of SP1 as the transcription factor through which SIA-cIgG regulates PTPN13, we found that both knockdown and overexpression of SP1 consistently enhanced cell sensitivity to drugs. This result reminds us that SP 1, as a transcription factor, regulates a broad network of genes beyond PTPN13, and its role in cell survival and drug response appears complex and likely context-dependent. Furthermore, this observation suggests that SP 1-mediated regulation of PTPN13 transcription likely involves changes in SP1 expression level. In transcriptional regulation, SP1 often requires the recruitment of other molecules to form functional complexes. For example, the acetyl transferase p300 can be recruited in an SP1-dependent manner to modulate genes like p16.^[50]^ Notably, p300 activity can be regulated by SRC/ERK pathway, which is activated by SIA-cIgG.^[51]^ Additionally, when multiple binding sites exist in a promoter, SP1 and SP3 can engage in competitive binding, leading to complex, context-specific transcriptional activation or repression.^[52]^ DNA methylation, a key mechanism implicated in cisplatin resistance, can also impair SP1 binding and promote gene silencing.^[53]^ Collectively, these factors indicate that the regulation of PTPN13 by SP1 is potentially influenced by a complex network, including co-factor recruitment, competitive interactions with other transcription factors, and epigenetic modifications. The precise mechanistic details require further investigation.

While many studies have reported the regulation of SP1 on downstream pathways, its own regulation by upstream factors is less understood. Although we could not fully elucidate this upstream cascade here, we have formulated two testable hypotheses based on prior literature and our data. At the transcriptional level, SIA-cIgG may promote NF-κB activation, which is known to suppress SP1 promoter activity.^[54]^ Chemotherapeutics can activate the canonical NF-κB pathway,^[55,56]^ wherein inhibitory IκB proteins are phosphorylated and degraded, releasing NF-κB transcription factors for nuclear translocation and target gene regulation.^[57]^ At the post-translational level, SIA-cIgG may regulate SP 1 protein stability *via* SOX2, which has been shown to promote SKP2 transcription and accelerate SP1 degradation.^[58]^ Therefore, we hypothesize that SIA-cIgG may regulate SP1 both by activating NF-κB-dependent transcriptional repression and by modulating SOX2-mediated protein stability. Future studies should investigate whether chemotherapy-induced NF-κB activation is SIA-cIgG-dependent and explore the role of the SIA-cIgG/ SOX2 axis in controlling SP1 stability.

Cancer stemness contributes significantly to intra- and inter-tumor heterogeneity, which in turn limits chemotherapy efficacy.^[27,36]^ Anti-SIA-cIgG-based drug combinations demonstrated superior tumor inhibition in 24/25 cases compared to dual-drug combinations, with 21/25 cases showing > 50% tumor viability reduction and 5/25 showing > 80% reduction. Compared to cetuximab-based drug combinations, anti-SIA-cIgG-based drug combinations were superior in 24/25 cases, achieving > 50% and > 80% viability reduction in 16 and 5 cases, respectively. These data indicate a significant anti-tumor advantage of anti-SIA-cIgG-based combination therapy over existing regimens. Overall, tumor viability was controlled below 40% of control levels in both anti-SIA-cIgG combination groups, with 22 of 25 PDOs falling below 30%. In contrast, viability varied widely in the other regimens (dual-drug combinations: 3.4%–131.81%; cetuximab-based drug combinations: 1.69%–95.69%), with three dual-drug- and one cetuximab-based therapy-treated PDOs even showing tumor progression. These results highlight the ability of anti-SIA-cIgG-based combination therapy to overcome drug response heterogeneity in drug response and underscore the potential of PDOs as a preclinical model for guiding clinical decisions. It is also noteworthy that RP215 monotherapy suppressed tumor viability by > 50% in 11 of 17 PDOs and by > 80% in 3 of 17 PDOs, although combination regimens performed better overall. This suggests that in selected cases, anti-SIA-cIgG monotherapy might achieve therapeutic goals while avoiding the added toxicity of combination chemotherapy. Furthermore, in xenograft models, RP215 suppressed tumor growth without affecting major organ histology or body weight, supporting the safety of targeting SIA-cIgG. While this study demonstrates a strong and consistent association between high SIA-cIgG expression and poor chemotherapy response, this link remains a predictive inference at present. Prospective studies correlating SIA-cIgG expression with patient outcomes would significantly strengthen the clinical relevance of our findings. Mechanistic studies would also benefit from establishing and analyzing PDOs derived from tumors both before and after chemotherapy. Finally, the development of a humanized anti-SIA-cIgG antibody represents a critical direction for future translational work.

**Figure 9:**
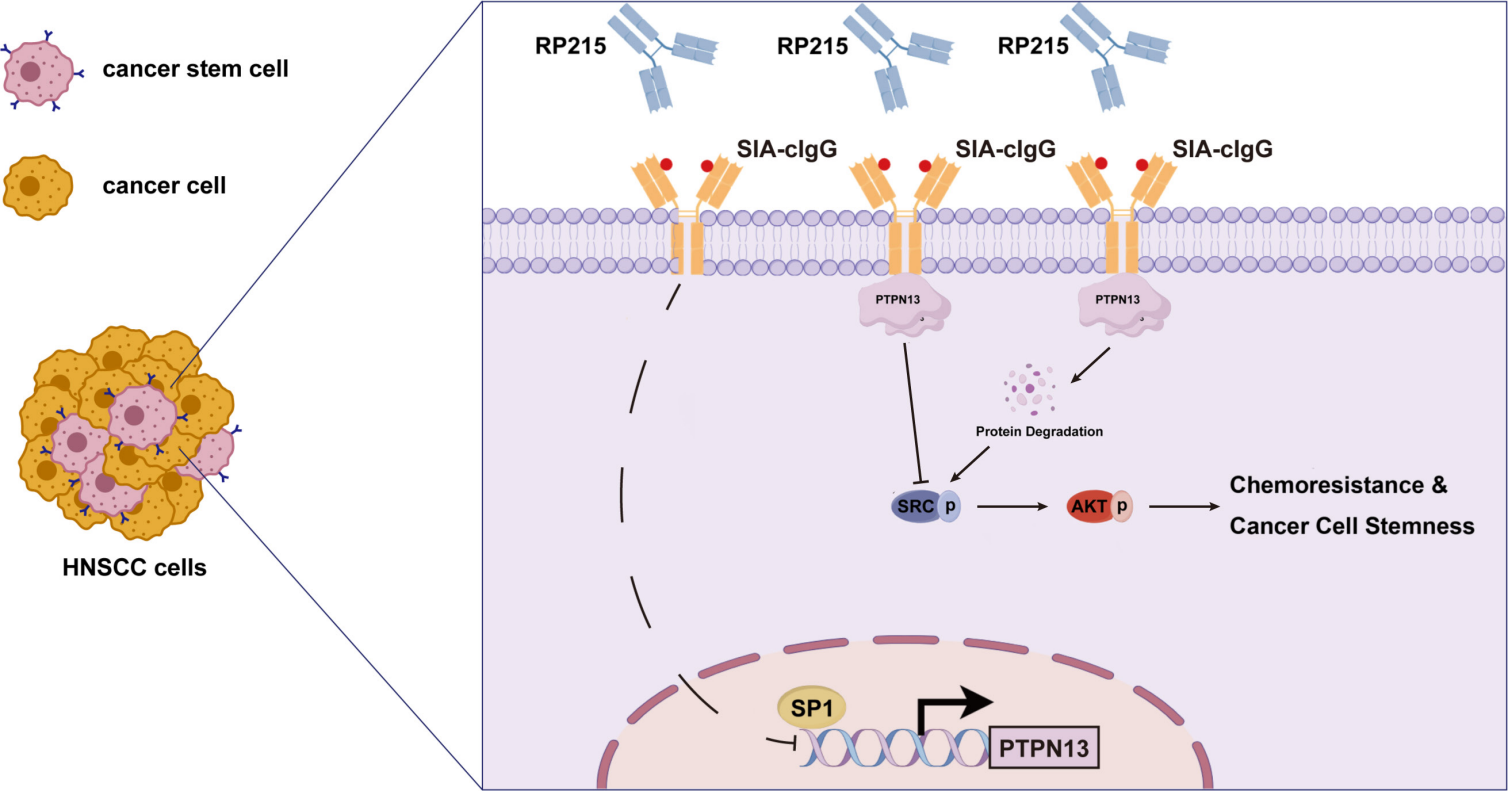
Schematic diagram of SIA-cIgG/PTPN13 axis in chemoresistance and tumor stemness (Generated by Figdraw).

In conclusion, this study establishes the SIA-cIgG/ PTPN13 axis as a critical therapeutic target in HNSCC, particularly for overcoming chemoresistance. The significantly superior antitumor efficacy of anti-SIA-cIgG-based combinations provides strong rationale for the clinical translation of humanized anti-SIA-cIgG antibodies and related combination strategies. A schematic summary of the mechanism is presented in Figure 9.

## Supplementary Material

Supplementary Material Details
