# Supplementary material for: Anti-SIA-cIgG enhances chemotherapy effectiveness through PTPN13-regulated tumor stemness in head and neck squamous cell carcinoma
Source: J Transl Int Med. 2026 Mar 26;14(2):237–58. doi: 10.1515/jtim-2026-0040 (PMC13110467; doi:10.1515/jtim-2026-0040)
Supplement: Supplementary file 1 — Supplementary Material Details [file jtim-2026-0040_sm.pdf]

Supplementary Materials for

Anti-Sialylated cancer IgG Enhances Chemotherapy Effectiveness Through PTPN13-regulated Cancer Cell Stemness in Head and Neck Squamous Cell Carcinoma

Running Title: Anti-SIA-cIgG in HNSCC Chemotherapy

This file includes: Supplementary Figure 1-10 and Supplementary Table 1-7

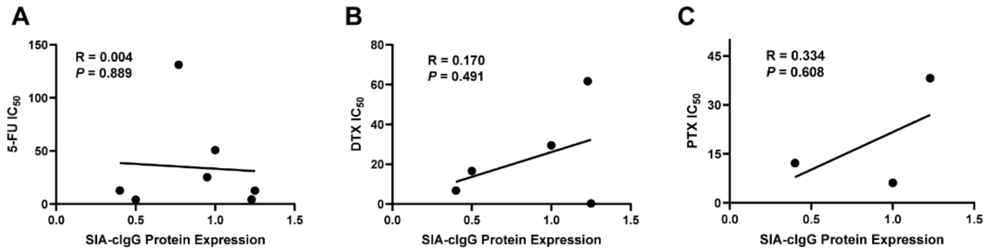

Supplementary Figure 1. Simple linear regression of SIA-cIgG protein expression and 5-FU, DTX, PTX. A Simple linear regression of SIA-cIgG protein expression and 5-FU IC_{50}, $n = 7$. **B** Simple linear regression of SIA-cIgG protein expression and DTX IC_{50}, $n = 5$. **C** Simple linear regression of SIA-cIgG protein expression and PTX IC_{50}, $n = 3$.

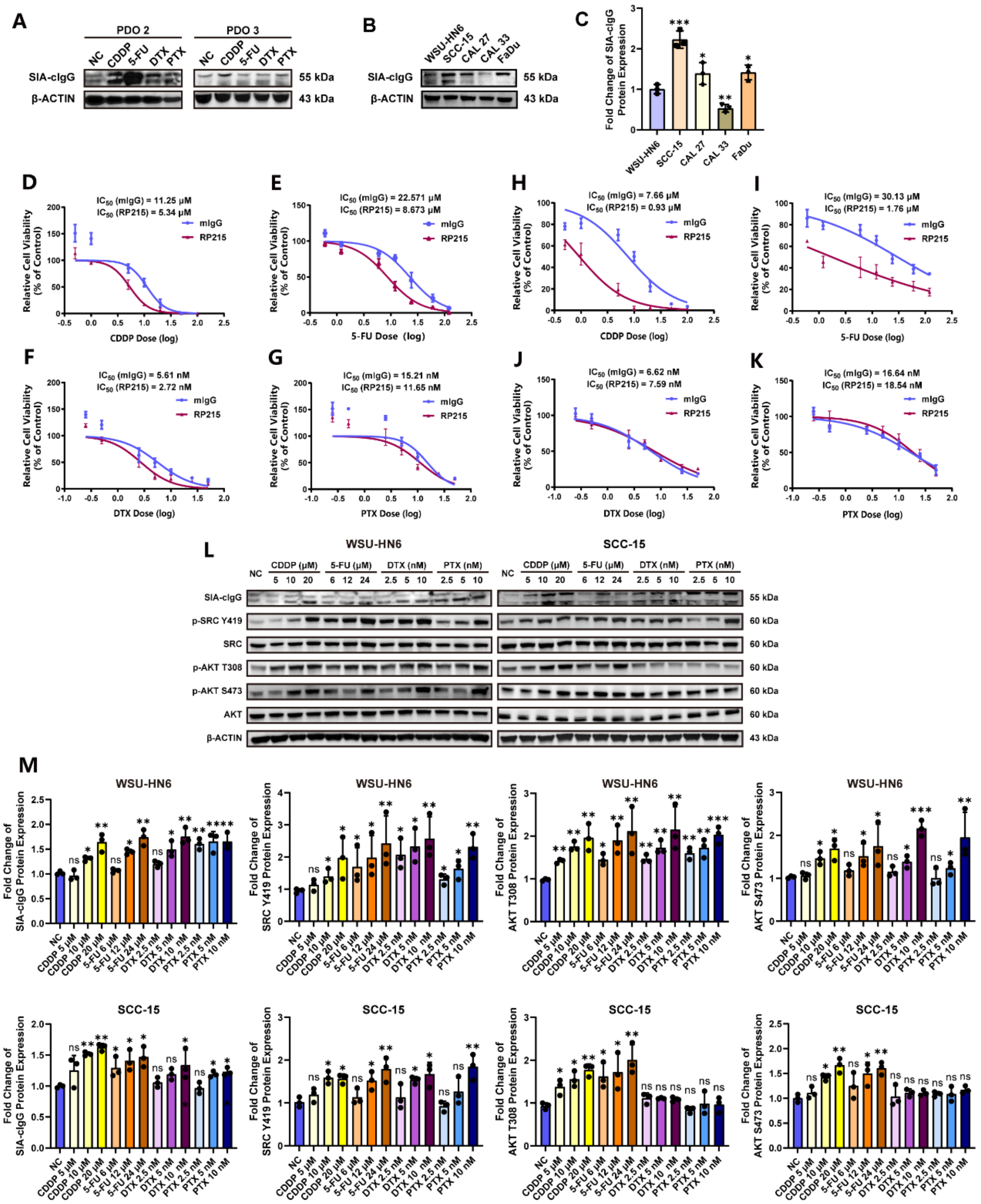
A
PDO 2
PDO 3
NC CDDP 5-FU DTX PTX
SIA-cIgG
β-ACTIN
55 kDa
43 kDa
B
WSU-HN6 SCC-15 CAL 27 CAL 33 FaDu
C
Fold Change of SIA-cIgG Protein Expression
D
IC50 (mIgG) = 11.25 μM
IC50 (RP215) = 5.34 μM
CDDP Dose (log)
E
IC50 (mIgG) = 22.571 μM
IC50 (RP215) = 8.673 μM
5-FU Dose (log)
H
IC50 (mIgG) = 7.66 μM
IC50 (RP215) = 0.93 μM
CDDP Dose (log)
I
IC50 (mIgG) = 30.13 μM
IC50 (RP215) = 1.76 μM
5-FU Dose (log)
F
IC50 (mIgG) = 5.61 nM
IC50 (RP215) = 2.72 nM
DTX Dose (log)
G
IC50 (mIgG) = 15.21 nM
IC50 (RP215) = 11.65 nM
PTX Dose (log)
J
IC50 (mIgG) = 6.62 nM
IC50 (RP215) = 7.59 nM
DTX Dose (log)
K
IC50 (mIgG) = 16.64 nM
IC50 (RP215) = 18.54 nM
PTX Dose (log)
Relative Cell Viability (% of Control)
mIgG
RP215
L
WSU-HN6
SCC-15
NC
CDDP (μM) 5 10 20
5-FU (μM) 6 12 24
DTX (nM) 2.5 5 10
PTX (nM) 2.5 5 10
SIA-cIgG 55 kDa
p-SRC Y419 60 kDa
SRC 60 kDa
p-AKT T308 60 kDa
p-AKT S473 60 kDa
AKT 60 kDa
β-ACTIN 43 kDa
M
WSU-HN6
SCC-15
Fold Change of SIA-cIgG Protein Expression
Fold Change of SRC Y419 Protein Expression
Fold Change of AKT T308 Protein Expression
Fold Change of AKT S473 Protein Expression
NC
CDDP 5 μM
CDDP 10 μM
CDDP 20 μM
5-FU 6 μM
5-FU 12 μM
5-FU 24 μM
DTX 2.5 nM
DTX 5 nM
DTX 10 nM
PTX 2.5 nM
PTX 5 nM
PTX 10 nM

N

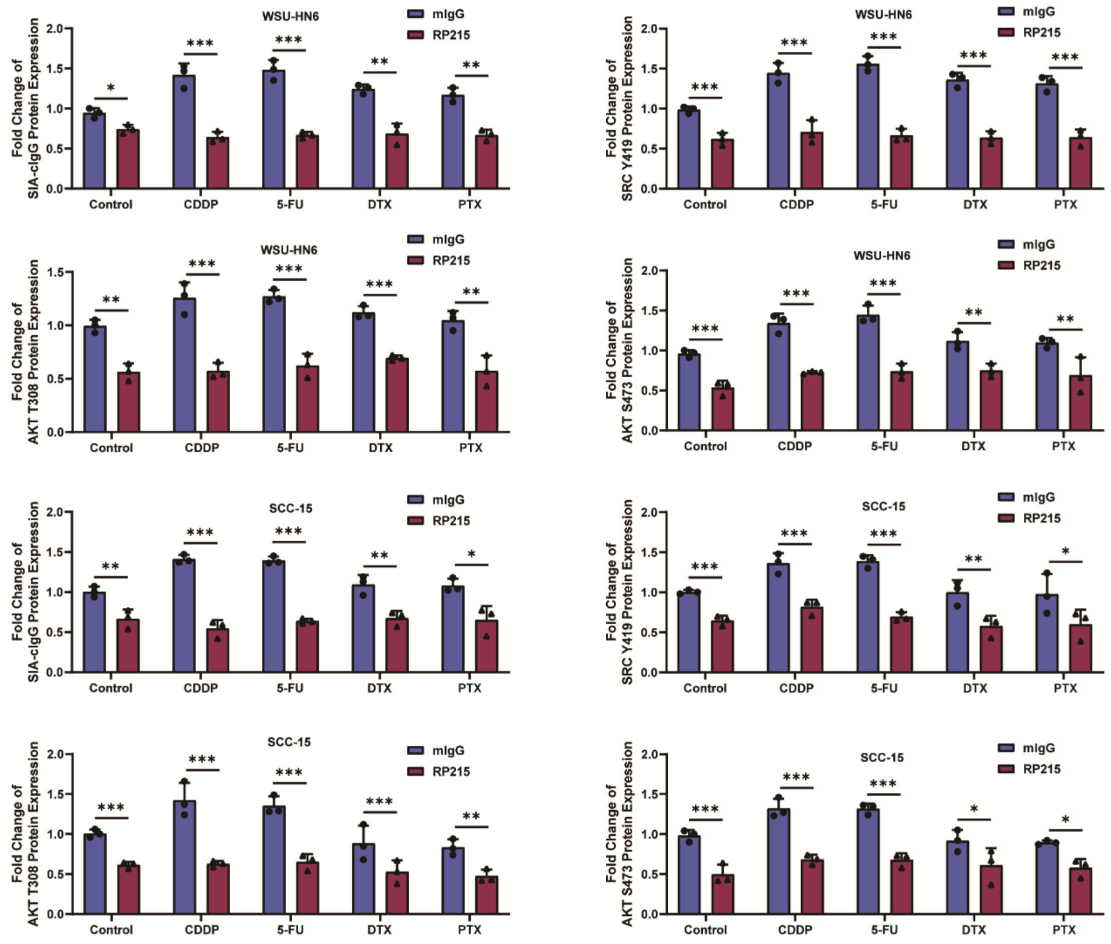

Supplementary Figure 2. A SIA-cIgG protein expression of PDO 2 and PDO 3 after different drug treatments for 6 days (medium and drugs renewed in day 4) by Western blot assay. **B** SIA-cIgG protein expression of 5 HNSCC cell lines by Western blot assay. **C** Fold change of SIA-cIgG protein expression in 6 HNSCC cell lines, $n = 3$ (respectively). **D-K** Drug response curves of CDDP, 5-FU, DTX and PTX in WSU-HN6 and SCC-15 with/without 20 μg/mL RP215. **D-G,** WSU-HN6; **H-K,** SCC-15. **L** SIA-cIgG, p-SRC Y419, SRC, p-AKT T308, p-AKT S473, and AKT protein expression after treatments of CDDP, 5-FU, DTX and PTX at different concentration for 48 hours in WSU-HN6 and SCC-15. **M** Fold change of SIA-cIgG, p-SRC Y419, p-AKT T308, and p-AKT S473 protein expression in Fig. S2L, $n = 3$ (respectively). **N**

Fold change of SIA-cIgG, p-SRC Y419, p-AKT T308, and p-AKT S473 protein expression in Fig. 2H, $n = 3$ (respectively). Data are represented as the mean ± SEM; *$P < 0.05$, **$P < 0.01$, ***$P < 0.001$, ns, no significant difference.

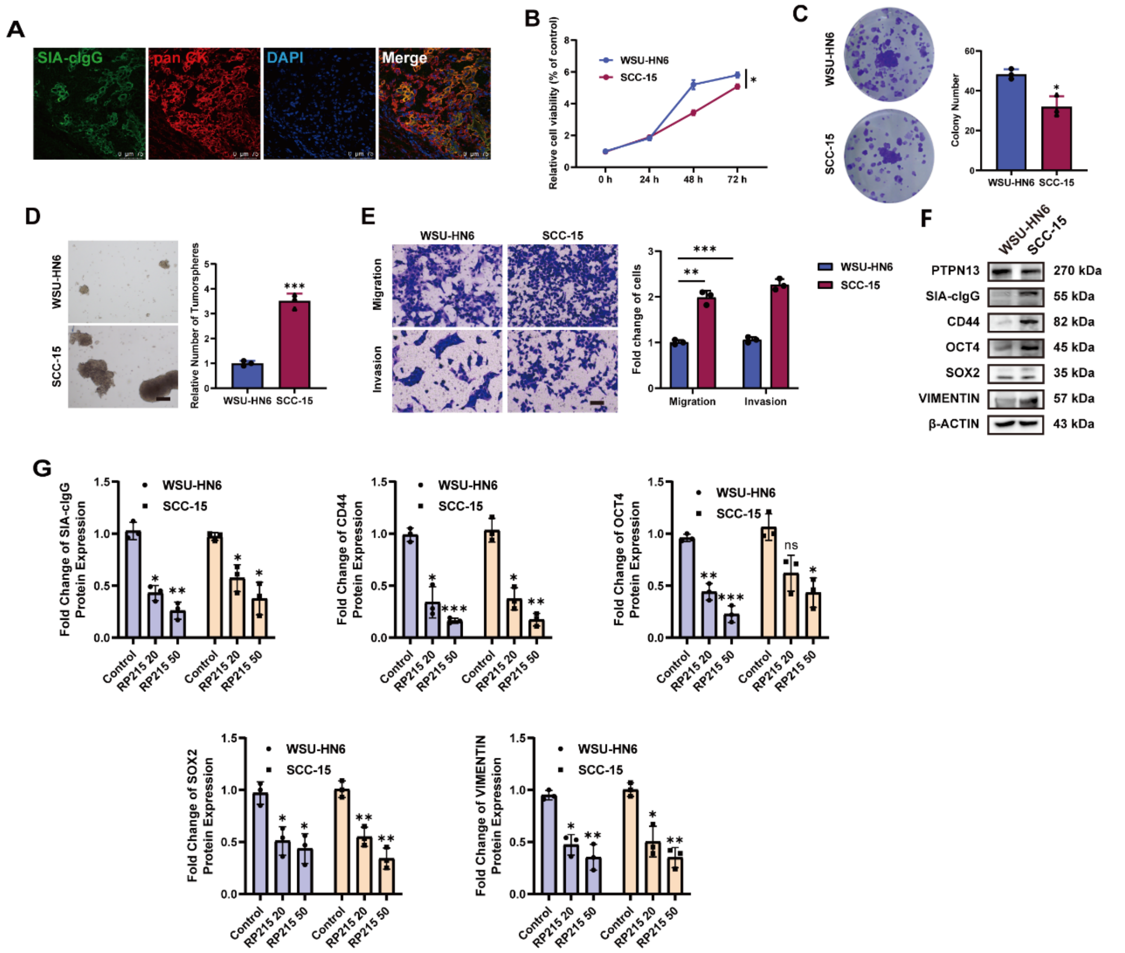

Supplementary Figure 3. A Representative fluorescence microphotographs of SIA-cIgG/pan CK staining in HNSCC tumor tissue. Scale bars, 75 μm. **B-E** Cell proliferation rates (**B**), Colony formation assay (**C**), *in vitro* tumorsphere formation assay (**D**), Transwell migration and invasion (**E**) of WSU-HN6 and SCC-15, $n = 3$ (respectively). Scale bars, **D,** 250 μm; **E,** 200 μm. **F** PTPN13, SIA-cIgG, CD44, OCT4, SOX2, and VIMENTIN protein expression in WSU-HN6 and SCC-15. **G** Fold change of SIA-cIgG, CD44, OCT4, SOX2 and VIMENTIN protein expression after 20 μg/mL or 50 μg/mL RP215 treatment for 48 hours, $n = 3$ (respectively). Data are represented as the mean ± SEM; $*P < 0.05$, $**P < 0.01$, $**P < 0.001$, ns, no significant difference.

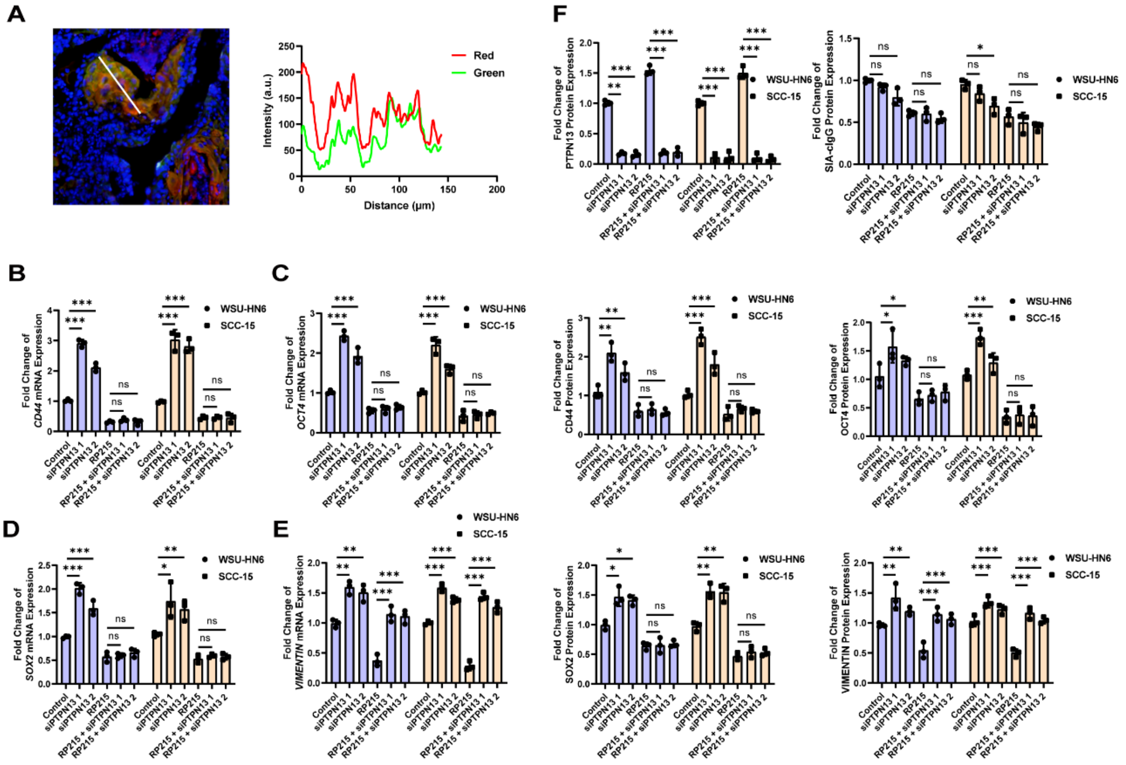

Supplementary Figure 4. A Co-localization analysis of SIA-cIgG/PTPN13 in tumor tissue. **B-E** CD44 (**B**), OCT4 (**C**), SOX2 (**D**) and VIMENTIN (**E**) mRNA expression of PTPN13 knockdown with/without 20 μg/mL RP215 treatment for 48 hours, n = 3 (respectively). **F** Fold change of PTPN13, SIA-cIgG, CD44, OCT4, SOX2, and VIMENTIN protein expression of PTPN13 knockdown with/without 20 μg/mL RP215 treatment for 48 hours, n = 3 (respectively). Data are represented as the mean ± SEM; *$P < 0.05$, **$P < 0.01$, ***$P < 0.01$, ns, no significant difference.

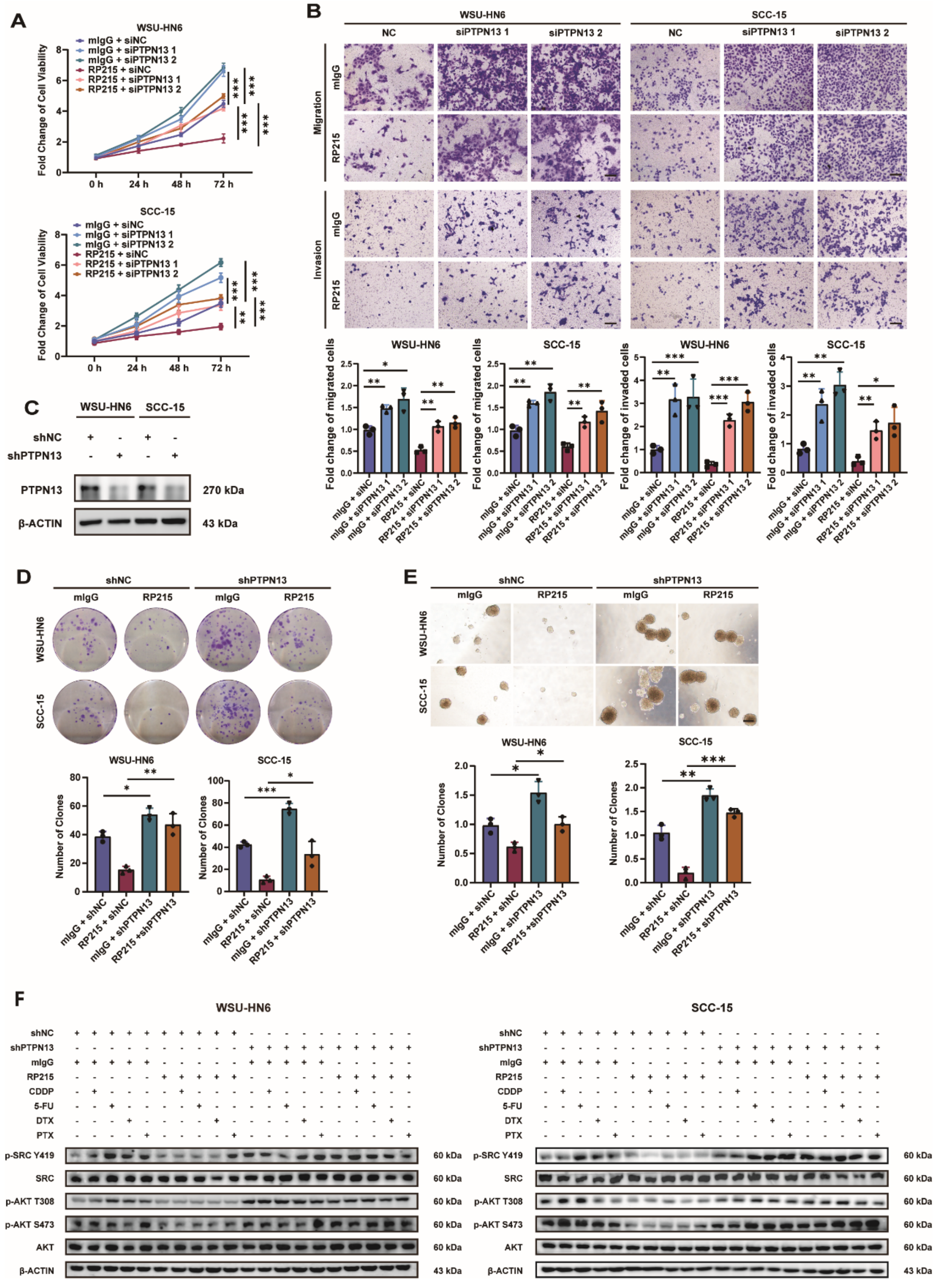
A
WSU-HN6
mIgG + siNC
mIgG + siPTPN13 1
mIgG + siPTPN13 2
RP215 + siNC
RP215 + siPTPN13 1
RP215 + siPTPN13 2
Fold Change of Cell Viability
0 h
24 h
48 h
72 h
SCC-15
B
WSU-HN6
SCC-15
NC
siPTPN13 1
siPTPN13 2
Migration
Invasion
mIgG
RP215
Fold change of migrated cells
Fold change of invaded cells
C
shNC
shPTPN13
PTPN13
β-ACTIN
270 kDa
43 kDa
D
E
Number of Clones
mIgG + shNC
RP215 + shNC
mIgG + shPTPN13
RP215 + shPTPN13
F
CDDP
5-FU
DTX
PTX
p-SRC Y419
SRC
p-AKT T308
p-AKT S473
AKT
60 kDa

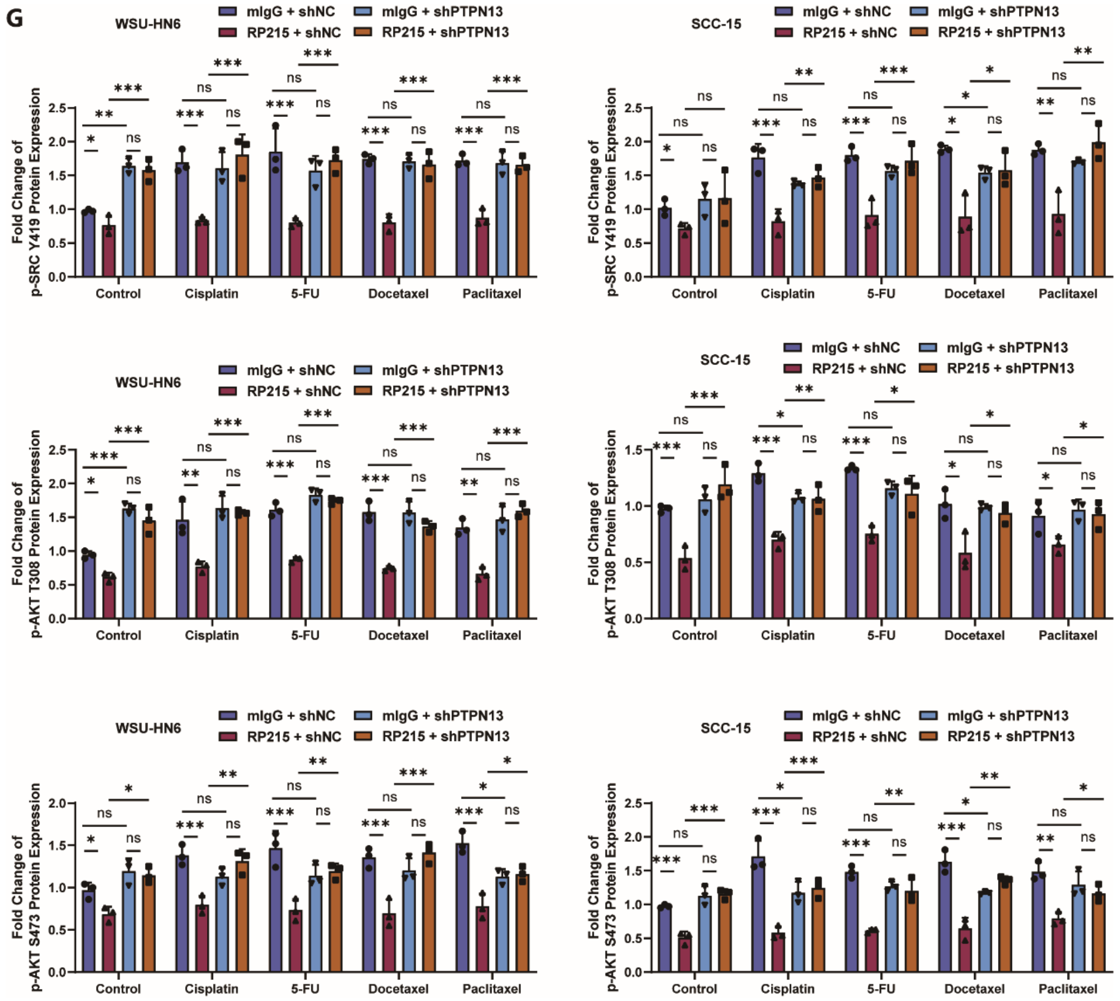

Supplementary Figure 5. A Cell proliferation rates of PTPN13 knockdown with/without 20 μg/mL RP215 treatment in WSU-HN6 and SCC-15, *n* = 3 (respectively). **B** Transwell migration and invasion of PTPN13 knockdown with/without 20 μg/mL RP215 treatment in WSU-HN6 and SCC-15. Scale bars, 200 μm. **C** Stable PTPN13 knockdown efficiency in WSU-HN6 and SCC-15 by Western blot. **D-E** Colony formation assay and *in vitro* tumorsphere formation assay of different PTPN13 status with/without 20 μg/mL RP215 treatment in WSU-HN6 and SCC-15, *n* = 3 (respectively). Scale bar for **E**, 250 μm. **F** p-SRC Y419, SRC, p-AKT T308, p-AKT S473, and AKT protein expression of WSU-HN6 and SCC-15 after different drug treatments with/without PTPN13 knockdown as well as 20 μg/mL RP215 treatment for

48 hours. **G** Fold change of p-SRC Y419, SRC, p-AKT T308, p-AKT S473, and AKT protein expression in Fig. S5F, $n = 3$ (respectively). Data are represented as the mean ± SEM; *$P < 0.05$, **$P < 0.01$, ***$P < 0.001$, ns, no significant difference.

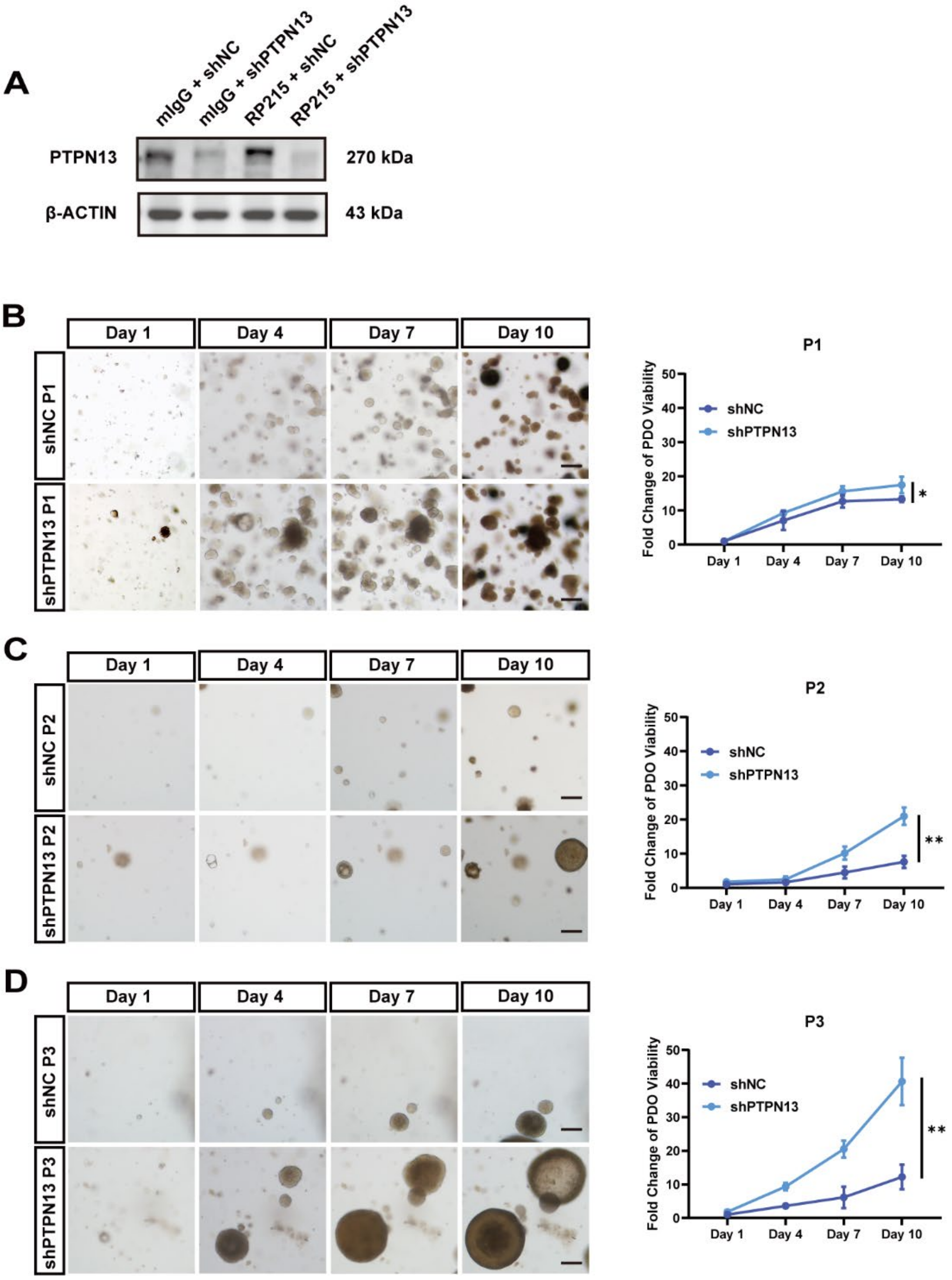

Supplementary Figure 6. A Stable PTPN13 knockdown efficiency in PDO by Western blot. **B-D** Representative microphotographs of continuous passage (P1, P2 and P3) at day 1, 4, 7,10 and proliferation rates of shNC or shPTPN13 HNSCC PDOs.

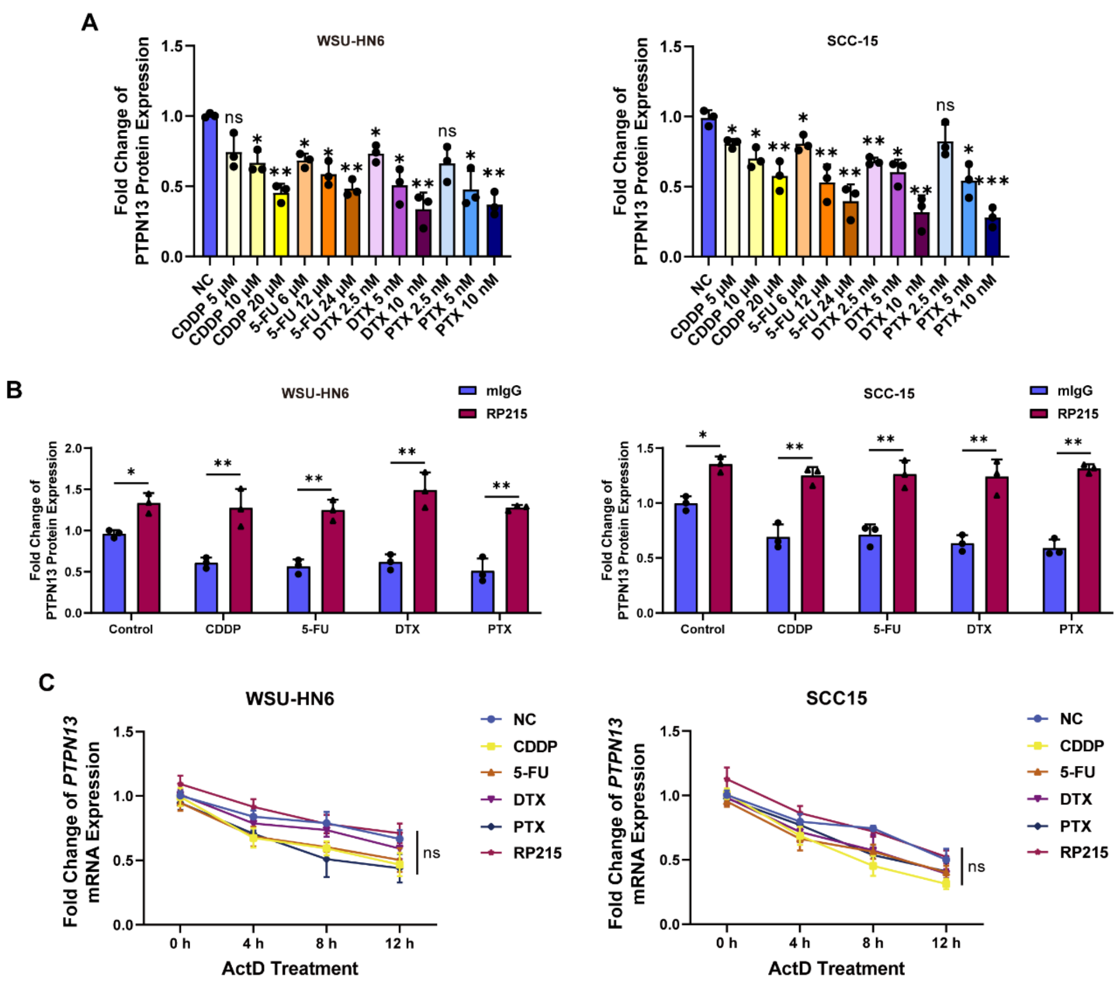

Supplementary Figure 7. A Fold change of PTPN13 protein expression in Fig. 5B, n = 3 (respectively). **B** Fold change of PTPN13 protein expression in Fig. 5D, n = 3 (respectively). **C** ActD assay for indicating PTPN13 mRNA half-life time. ActD: 2 μmol/L, n = 3 (respectively). Data are represented as the mean ± SEM; *$P < 0.05$, **$P < 0.01$, ***$P < 0.01$, ns, no significant difference.

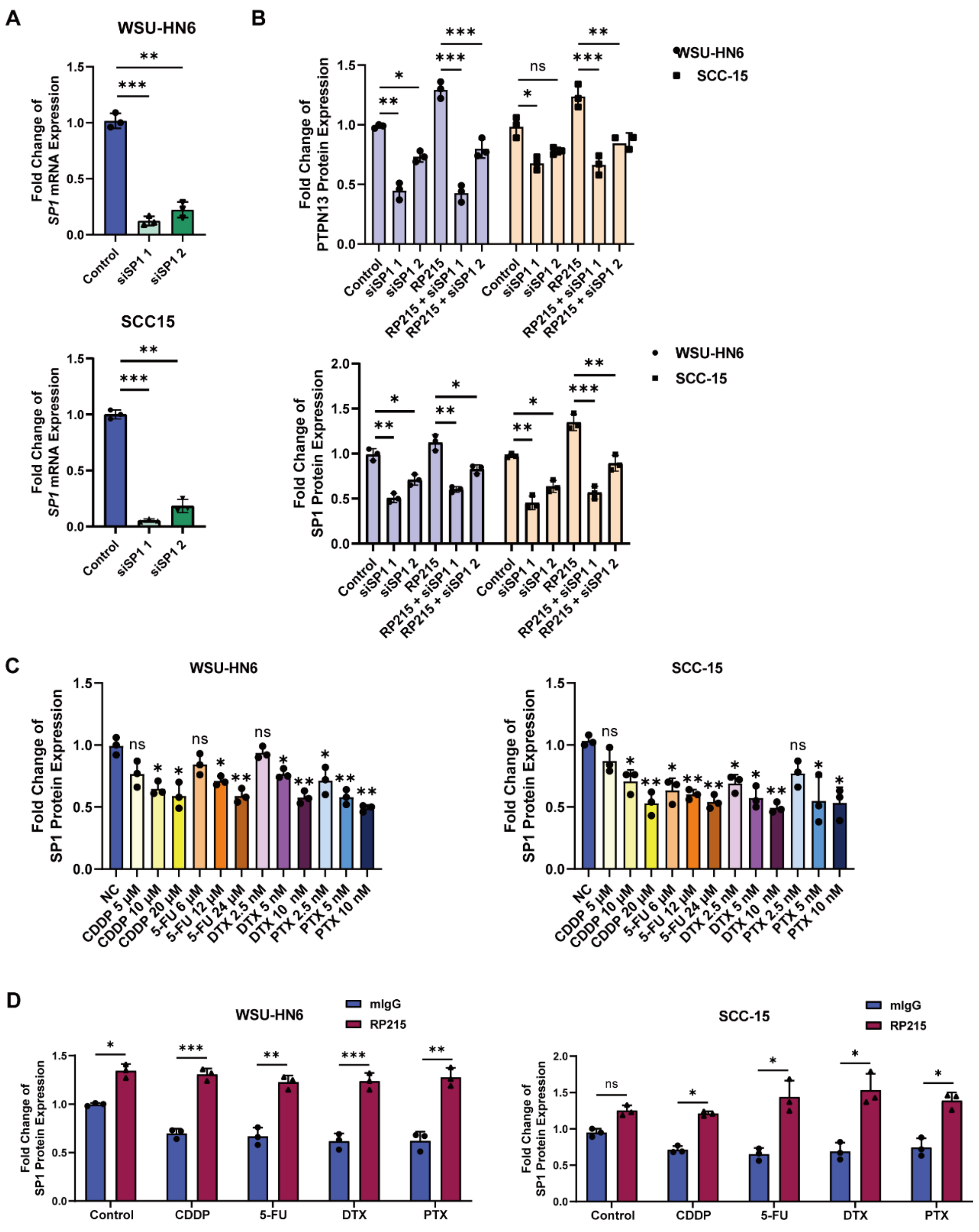

Supplementary Figure 8. A SP1 knockdown efficacy in WSU-HN6 and SCC-15. **B** Fold change of PTPN13 and SP1 protein expression in Fig. 6F, n = 3 (respectively). **C** Fold change of SP1 protein expression in Fig. 6H, n = 3 (respectively). **D** Fold change of PTPN13 protein expression in Fig. 6I, n = 3 (respectively). Data are represented as the mean ± SEM; *$P < 0.05$, **$P < 0.01$, ***$P < 0.01$, ns, no significant difference.

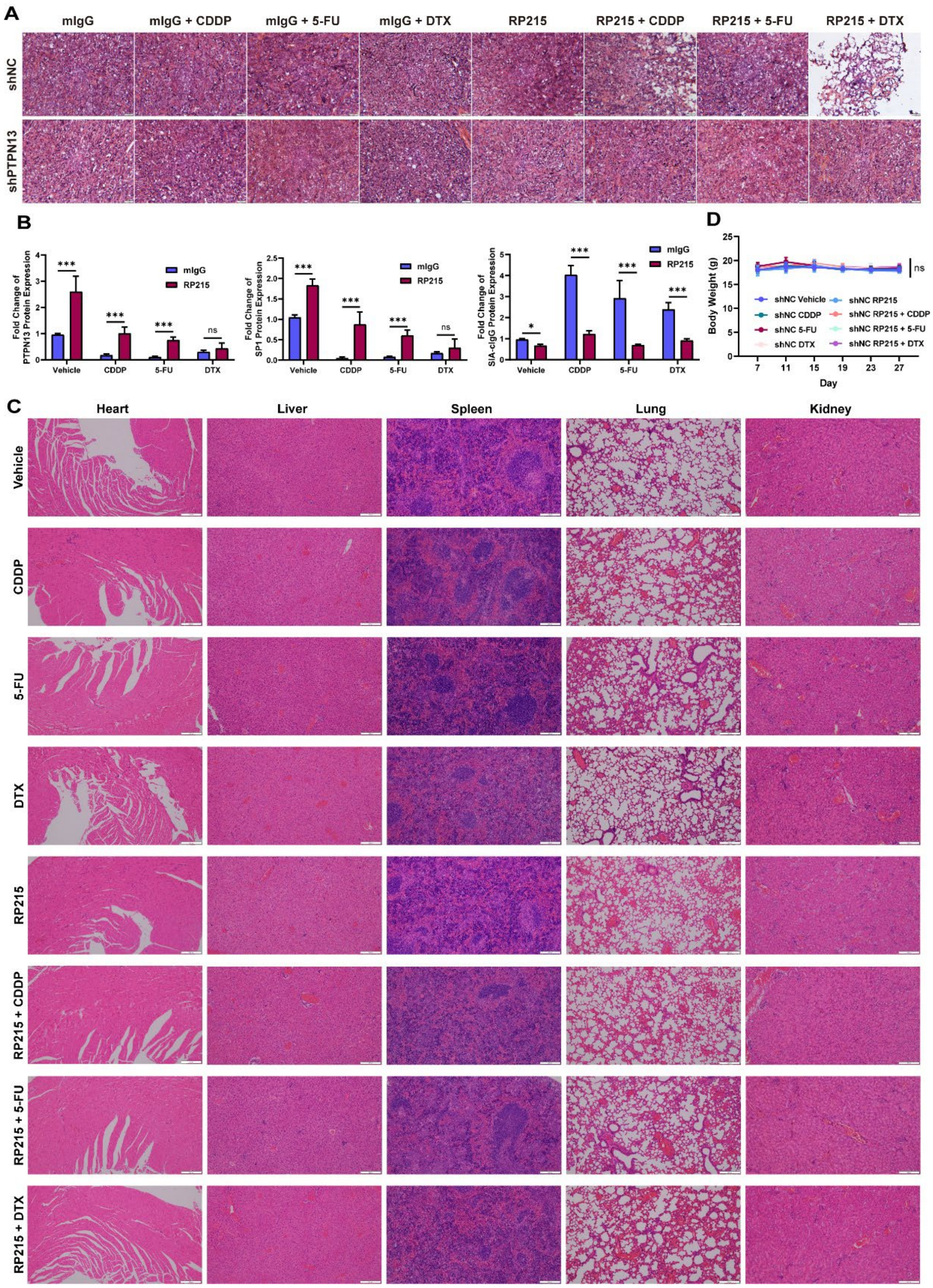

Supplementary Figure 9. A Representative H&E staining of xenograft tumors. **B** Fold change of PTPN13 SP1 and SIA-cIgG protein expression in Fig. 7N, n = 3 (respectively). **C** Representative H&E staining of major organs (heart, liver, spleen,

lung, kidney) from mice after different drugs treatment. **D** Line graph depicting body weight changes in experimental mice over time. ns, no significant difference.

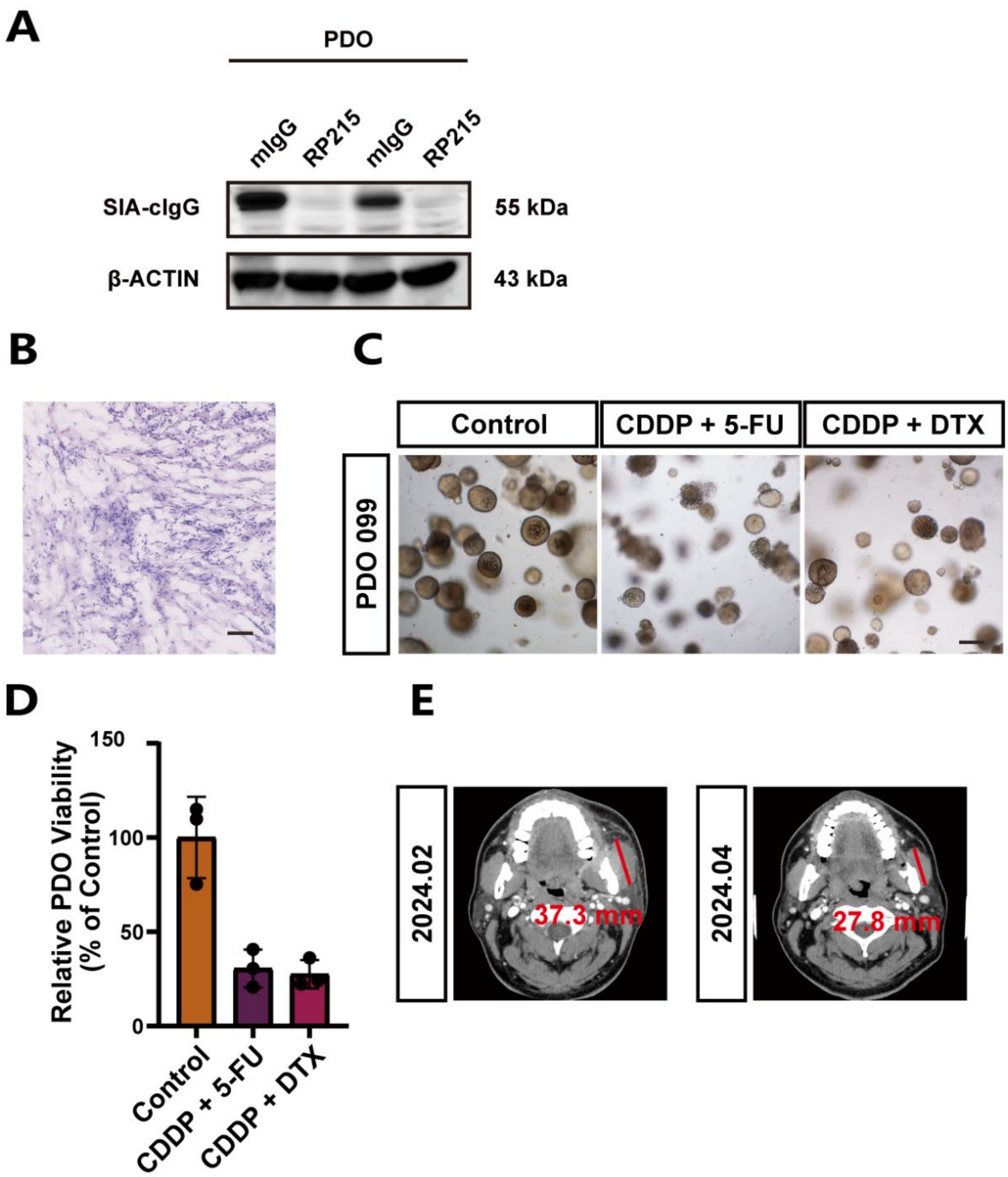

Supplementary Figure 10. PDO with low SIA-cIgG expression showed reduced organoid size and viability after treatment, accompanied by partial tumor regression on CT. **A** SIA-cIgG protein expression levels in PDOs following RP215 neutralization. **B** Representative microphotographs of SIA-cIgG IHC staining in patient 099 tumor tissue**,** Scale bar, 50 μm. **C** Representative microphotographs of PDO 099 after different treatments. Scale bar, 250 μm. **D** Relative PDO viability (% of control) of PDO 099 after different drug combination treatments. **E** Pre- and post-TPF

chemotherapy CT images of patient 099.

Supplementary Table 1. Clinical characteristics of 56 HNSCC patients in IHC and Western blot tests

Characteristics	Number of Cases（%)
Age	
< 60	32 (57.1)
≥ 60	24 (42.9)
Gender	
Female	16 (28.6)
Male	40 (71.4)
Site	
Tongue	28 (50)
Buccal mucosa	8 (14.3)
Gingiva	13 (23.2)
Floor of mouth	5 (8.9)
Jaw	2 (3.6)
T stage	
T1	5 (8.9)
T2	23 (41.1)
T3	13 (23.2)
T4	15 (26.8)
Lymph invasion	
Positive	35 (62.5)
Negative	21 (37.5)
Distant metastasis	
M0	56 (100)
M1	0 (0)

Supplementary Table 2. Clinical characteristics of 25 patients in PDO establishment

No.	Age	Gender	Site	T stage	Lymph invasion
044	60	Male	Jaw	T1	N1
048	66	Female	Gingiva	T2	N2
049	67	Male	Tongue	T3	N2
053	31	Male	Tongue	T2	N0
054	62	Female	Jaw	T3	N0
055	53	Female	Gingiva	T2	N1
057	59	Male	Tongue	T3	N0
061	64	Male	Tongue	T2	N0
062	59	Male	Tongue	T2	N0
066	64	Male	Floor of mouth	T2	N0
068	34	Male	Tongue	T2	N0
083	61	Male	Tongue	T2	N0
089	55	Male	Gingiva	T4	N1
090	65	Female	Tongue	T1	N1
094	61	Female	Gingiva	T4a	N2
098	33	Male	Tongue	T3	N1
099	48	Male	Gingiva	T4	N2
100	60	Female	Gingiva	T2	N1
101	48	Male	Gingiva	T4a	N2
107	54	Male	Floor of mouth	T1	N2
111	32	Male	Tongue	T2	N2
112	75	Female	Buccal mucosa	T4a	N1
113	58	Male	Tongue	T2	N0
115	46	Male	Tongue	T2	N2
116	66	Male	Tongue	T3	N1

Supplementary Table 3. Antibody information

Antibodies	Company & Cat.	Application
RP 215	Santa Cruz #sc-69849	1:200 for IHC and IF
		1:1000 for WB
		1:20 for IP
PTPN13	Proteintech #25944-1-AP	1:150 for IF
		1:1000 for WB
		1:50 for IP
SP1	Proteintech #21962-1-AP	1:4000 for WB
β-actin	ABclonal #AC004	1:5000 for WB
p-AKT T308	Cell Signaling Technology #9275S	1:500 for WB
p-AKT S473	ABclonal #AP1453	1:1000 for WB
AKT	ABclonal #A22412	1:1000 for WB
p-SRC Y419	ABclonal #AP1027	1:1000 for WB
SRC	Cell Signaling Technology #2108S	1:1000 for WB
CD44	Abcam #ab30436	1:200 for IF
		1:1000 for WB
SOX2	ABclonal #A11501	1:200 for IF
		1:1000 for WB
OCT4	ABclonal #A7920	1:200 for IF
		1:1000 for WB
Vimentin	Abcam #ab92547	1:200 for IF
		1:1000 for WB
pan Cytokeratin	Abcam #ab7753	1:200 for IF
HRP-conjugated goat anti-rabbit secondary antibody	ZSGB-BIO #ZB-2301	1:10000 for WB
HRP-conjugated goat anti-rabbit secondary antibody	ZSGB-BIO #ZB-2305	1:10000 for WB
FITC-conjugated goat anti-mouse secondary antibody	ZSGB-BIO #ZF-0312	1:200 for IF
Alexa Fluor® 594-conjugated goat anti-mouse antibody	ZSGB-BIO #ZF-0513	1:200 for IF
FITC-conjugated goat anti-rabbit secondary antibody	ZSGB-BIO #ZF-0311	1:200 for IF
Alexa Fluor® 594-conjugated goat anti-rabbit antibody	ZSGB-BIO #ZF-0516	1:200 for IF

Supplementary Table 4. Primer and siRNA

Gene	Sequences (5'-3')	Assay
β-ACTIN	Forward: CATGTACGTTGCTATCCAGGC<br>Reverse: CTCCTTAATGTCACGCACGAT	qPCR
IGHGc	Forward: ACTACAAGACCACGCCTCC<br>Reverse: CGTCGCACTCATTTACCC	qPCR
PTPN13	Forward: TTGGAATGACACTGTATTGGGG<br>Reverse: CCAAGCAGTATGCTGTTGAGAT	qPCR
SP1	Forward: CACCAGAATAAGAAGGGAGG<br>Reverse: GGTGGTAATAAGGGCTGAA	qPCR
CD44	Forward: GAGACAGCAACCAAGAGGCA<br>Reverse: GTGTGGTTGAAATGGTGCTG	qPCR
OCT4	Forward: GGGAGATTGATAACTGGTGTGTT<br>Reverse: GTGTATATCCCAGGGTGATCCTC	qPCR
SOX2	Forward: TGGACAGTTACGCGCACAT<br>Reverse: CGAGTAGGACATGCTGTAGGT	qPCR
VIMENTIN	Forward: CAGATGCGTGAAATGGAAGA<br>Reverse: TCCAGCAGCTTCCTGTAGGT	qPCR
siPTPN13-1	Forward: CCCUCAUUUUCCUACGUGATT<br>Reverse: UCACGUAGGAAAAUGAGGGTT	Knockdown
siPTPN13-2	Forward: GGAAAGAAGAGUUCGUUUATT<br>Reverse: UAAACGAACUCUUCUUUCCTT	Knockdown
siSP1-1	Forward: UGGUGGUGAUGGAAUACAUTT<br>Reverse: AUGUAUUCCAUCACCACCATT	Knockdown
siSP1-2	Forward: GCAAGUUCUGACAGGACUACCTT<br>Reverse: GGUAGUCCUGUCAGAACUUGCTT	Knockdown
PTPN13 Promoter-1	Forward: GGCGACCTCGTCCCTAGGGA<br>Reverse: GGCTCGCCAGCACGACGGT	ChIP-qPCR
PTPN13 Promoter-2	Forward: CCTACTCAAAGTTTGGCTGCAC<br>Reverse: TAAGCTAAGTCCCCGCTCCC	ChIP-qPCR
PTPN13 Promoter-3	Forward: CGTTGACCCCGCCATTTCTA<br>Reverse: CTCCCTTACTCTTCGCTGCC	ChIP-qPCR

Supplementary Table 5 IC_{50} of CDDP, 5-FU, DTX, PTX in 7 HNSCC PDOs

No.	SIA-cIgG Expression	CDDP (μM)	5-FU (μM)	DTX (μM)	PTX (μM)
089	1	4.66	50.78	29.51	6.13
090	1.25	4.09	12.73	0.31	-
094	0.5	2.67	4.11	16.63	-
098	1.23	6.71	4.33	61.72	38.20
099	0.4	0.84	12.80	6.83	12.19
100	0.77	4.58	131.20	-	-
101	0.95	7.72	25.29	-	-

Supplementary Table 6 Relative Cell Viabilities After RP215 Treatment in 17 HNSCC PDOs

No.	RP215 (% of Control)
049	10.2
053	54.46
054	6.76
055	20.49
057	36.99
061	61.41
062	27.64
066	22.08
068	17.58
089	62.22
090	23.37
098	41.54
100	55.36
107	48.53
111	53.39
112	33.22
116	41.44

Supplementary Table 7 25 PDOs Viabilities (% of Control) After Different Treatments

No.	CDDP + 5-FU	CDDP + DTX	CDDP + 5-FU + CTX	CDDP + DTX + CTX	CDDP + 5-FU + RP215	CDDP + DTX + RP215
044	15.07	18.38	14.45	7.58	6.39	5.68
048	9.5	13.1	10.8	4.67	5.26	3.21
049	20.14	11.31	12.67	6.16	5.92	1.59
053	45.26	36.75	43.64	34.46	18.8	22.98
054	7.46	7.33	4.82	8.09	3.43	2.93
055	54.3	30.44	28.09	34.33	24.22	19.16
057	42.72	37.9	25.3	45.25	11.66	22.39
061	71.7	84.56	75.06	45.97	17	21.24
062	67.64	59.35	83.66	81.78	6.39	3.05
066	29.79	19.54	22.3	30.15	6.5	8.2
068	46.07	30.29	27.47	11.82	7.04	6.39
083	97.38	84.15	91.52	71.86	33.51	28.76
089	39.41	48.33	27.68	23.86	16.21	24.67
090	28.58	12.43	18.36	5.36	30.8	11.44
094	26.97	20.29	19.76	10.37	4.14	3.63
098	57.76	87.3	95.69	81.16	2.27	3.31
099	30.6	35.16	26.85	24.54	12.14	19.52
100	94.72	130.41	80.84	80.81	2.35	6.07
101	92.27	131.81	55.38	59.81	4.93	7.55
107	107.73	112.85	50.03	54.89	38.97	23.46
111	6.2	3.4	2.3	1.69	0.7	0.52
112	60.45	32.44	42.76	43.28	4.62	1.94
113	41.31	67.31	47.8	52.22	3.48	3.34
115	12.4	37.6	9.2	27.96	2.76	1.93
116	41.46	50.2	37.63	38.75	26.05	22.36
